# Supplementary material for: A Naturally Occurring Urinary Collagen Type I Alpha 1-Derived Peptide Inhibits Collagen Type I-Induced Endothelial Cell Migration at Physiological Concentrations
Source: Int J Mol Sci. 2025 Aug 2;26(15):7480. doi: 10.3390/ijms26157480 (PMC12347341; doi:10.3390/ijms26157480)
Supplement: Supplementary file 1 [file ijms-26-07480-s001.zip › Supplementary Figures.pdf]

Supplementary Figures

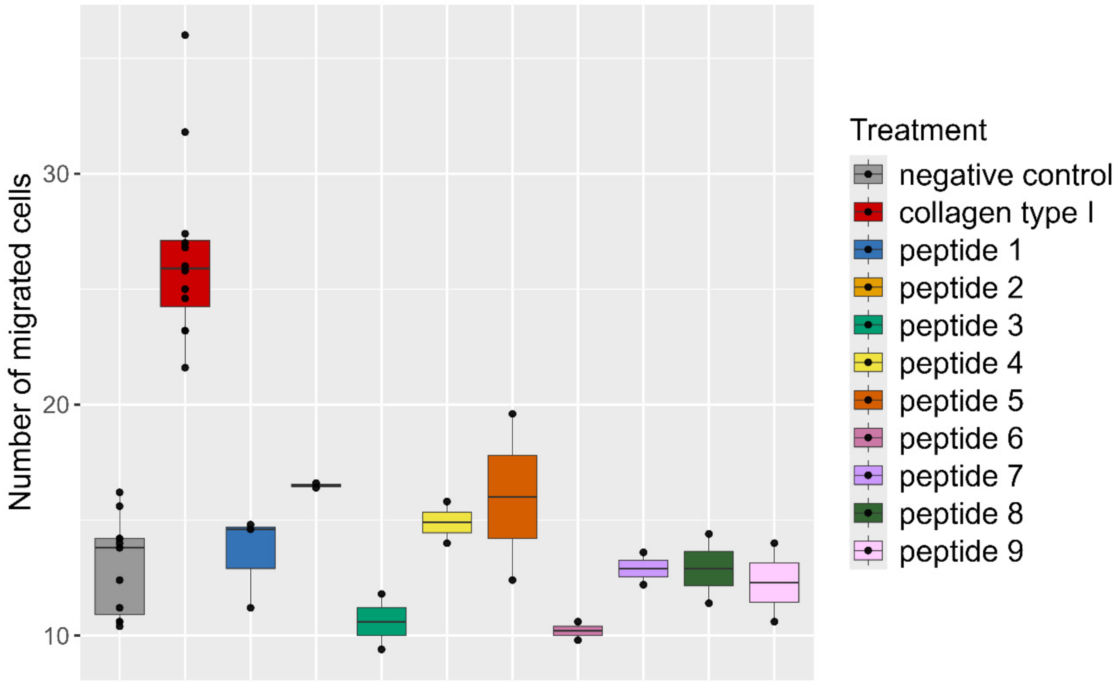

Supplementary Figure S1: boxplots of the average number of migrated cells per biological replicate, per cell treatment for matrikine-treated cells (n= 2, except for matrikine 1 where n=3). Results for collagen type I-treated cells (n=12) and negative control (n=11) treated cells are shown for reference.

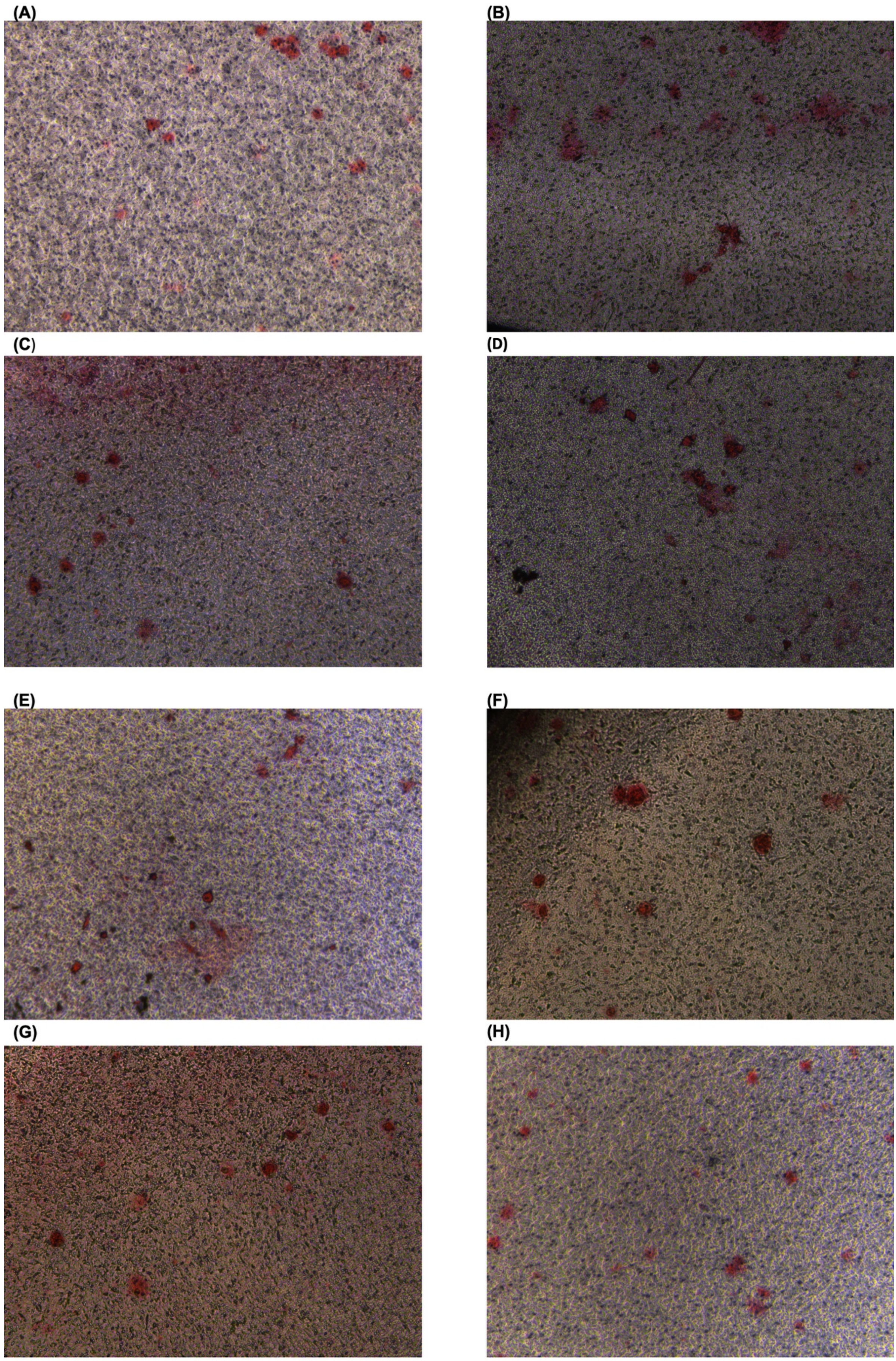
(A)
(B)
(C)
(D)
(E)
(F)
(G)
(H)

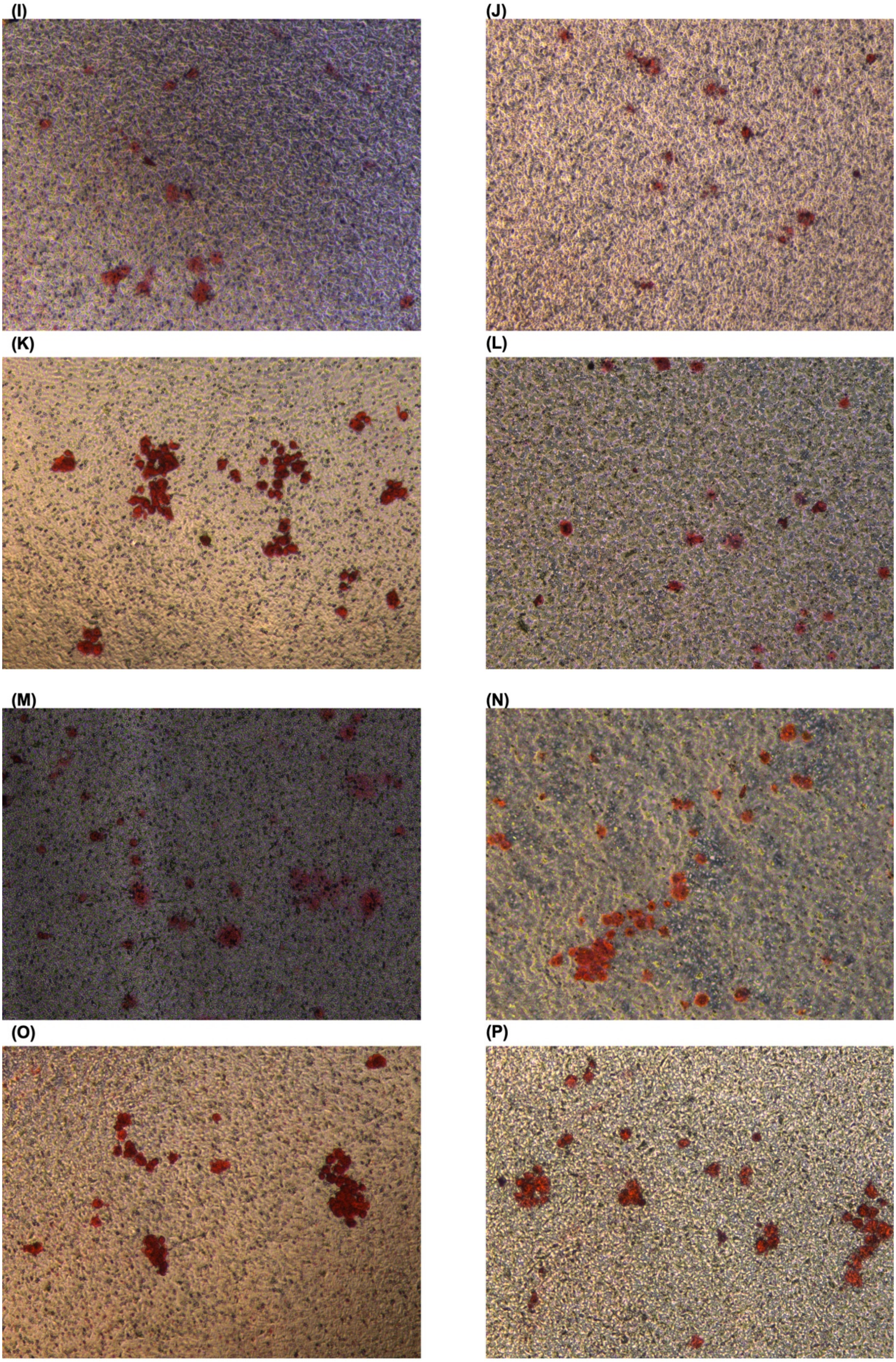
(I)
(J)
(K)
(L)
(M)
(N)
(O)
(P)

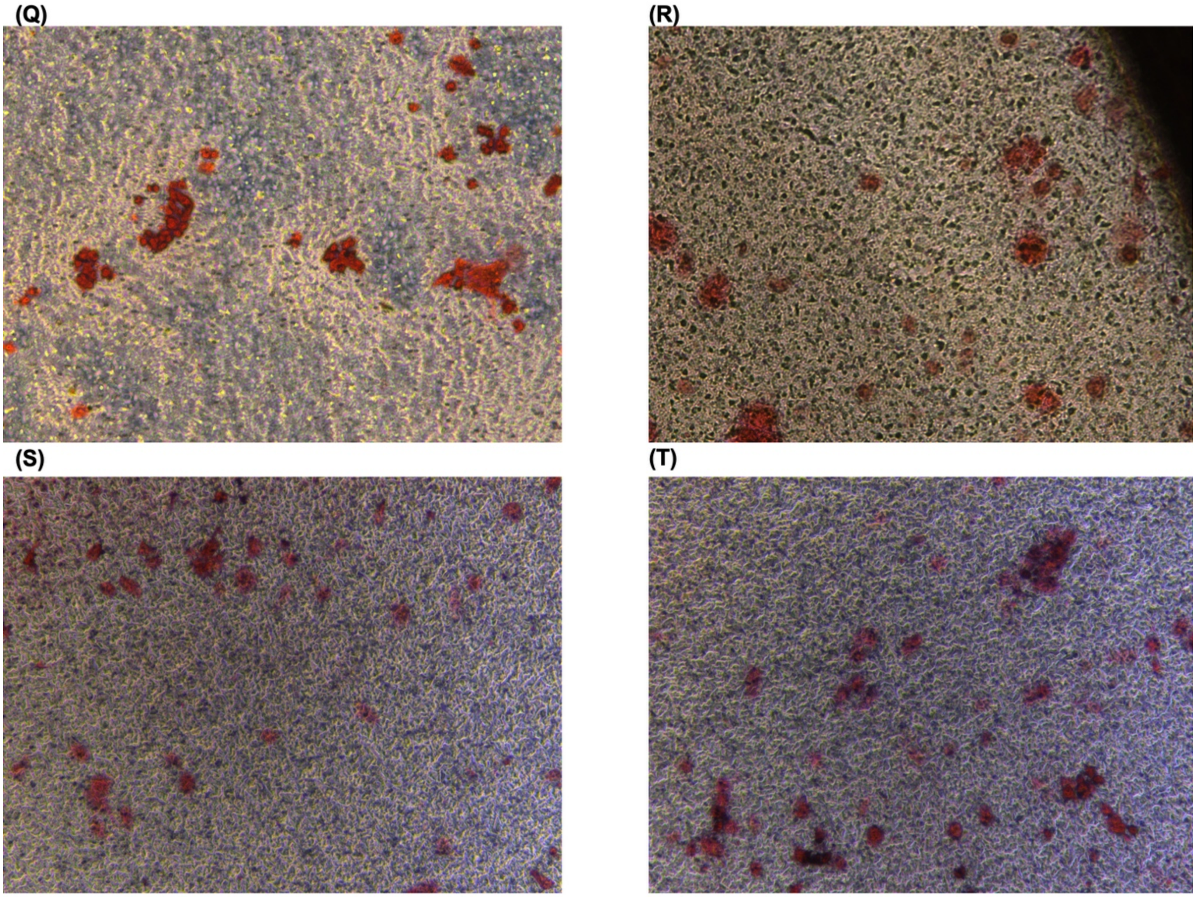

Supplementary Figure S2: Representative images of transwell inserts in the migration assays. A-I: matrikine 1-9 respectively, J: negative control, K: collagen type I, L-T: matrikine 1-9 + collagen type I respectively. Images taken at 10X magnification.

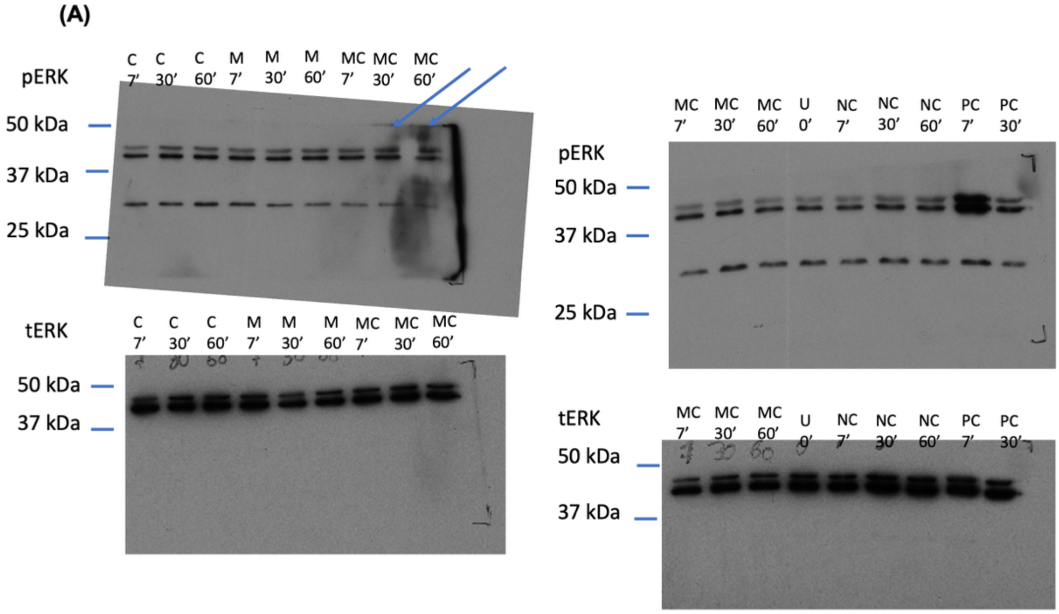

(B)

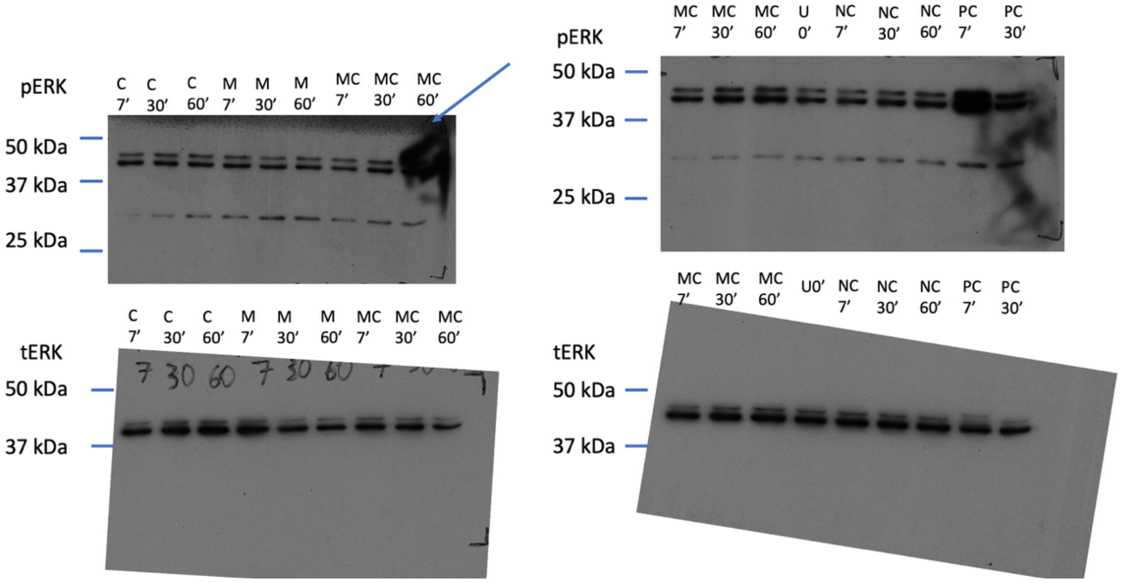

(C)

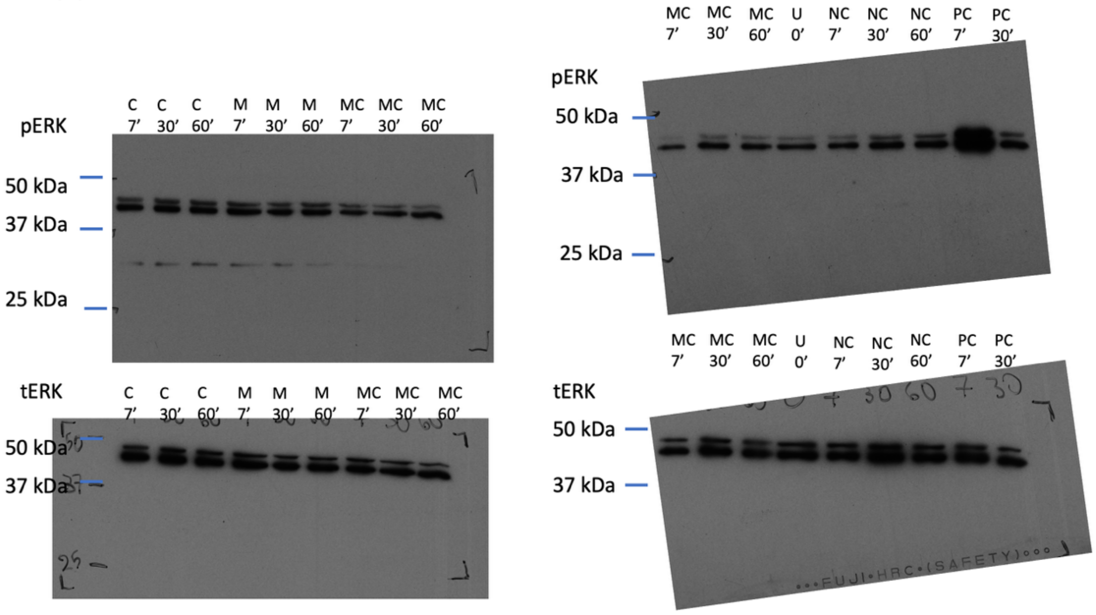

(D)

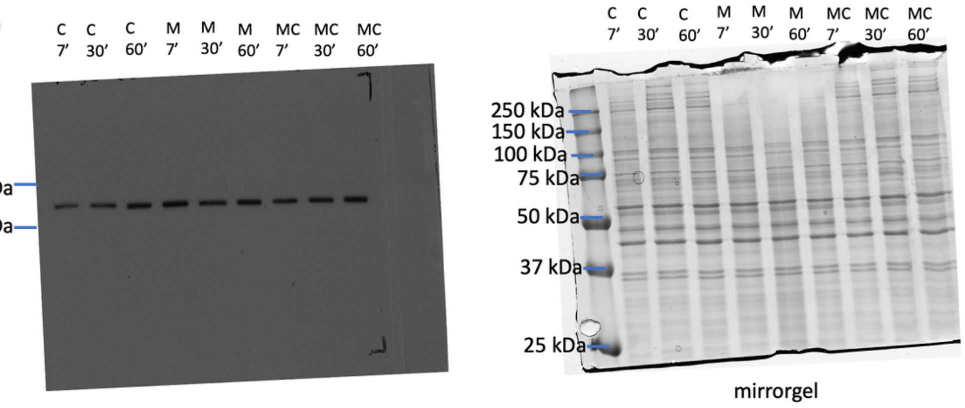

mirrorgel

(E)

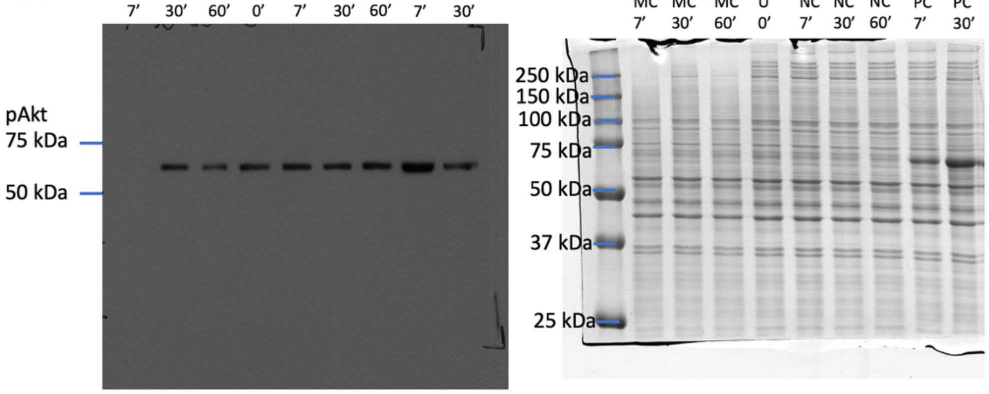
MC 7' MC 30' MC 60' U 0' NC 7' NC 30' NC 60' PC 7' PC 30'
pAkt
75 kDa
50 kDa
MC 7' MC 30' MC 60' U 0' NC 7' NC 30' NC 60' PC 7' PC 30'
250 kDa
150 kDa
100 kDa
75 kDa
50 kDa
37 kDa
25 kDa

(F)

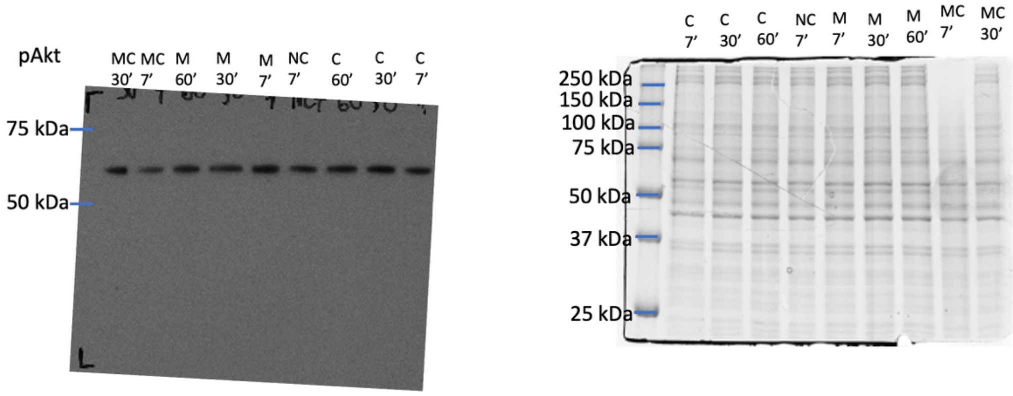
pAkt
MC 30' MC 7' M 60' M 30' M 7' NC 7' C 60' C 30' C 7'
75 kDa
50 kDa
C 7' C 30' C 60' NC 7' M 7' M 30' M 60' MC 7' MC 30'
250 kDa
150 kDa
100 kDa
75 kDa
50 kDa
37 kDa
25 kDa

(G)

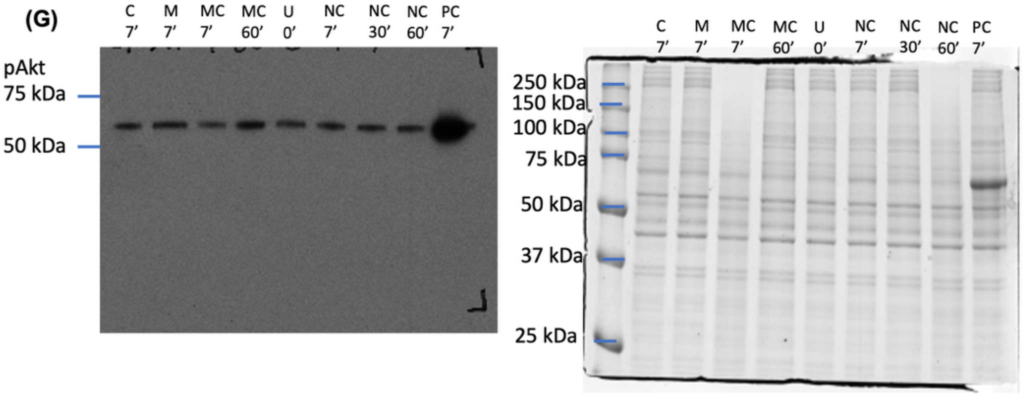
C 7' M 7' MC 7' MC 60' U 0' NC 7' NC 30' NC 60' PC 7'
pAkt
75 kDa
50 kDa
C 7' M 7' MC 7' MC 60' U 0' NC 7' NC 30' NC 60' PC 7'
250 kDa
150 kDa
100 kDa
75 kDa
50 kDa
37 kDa
25 kDa

(H)

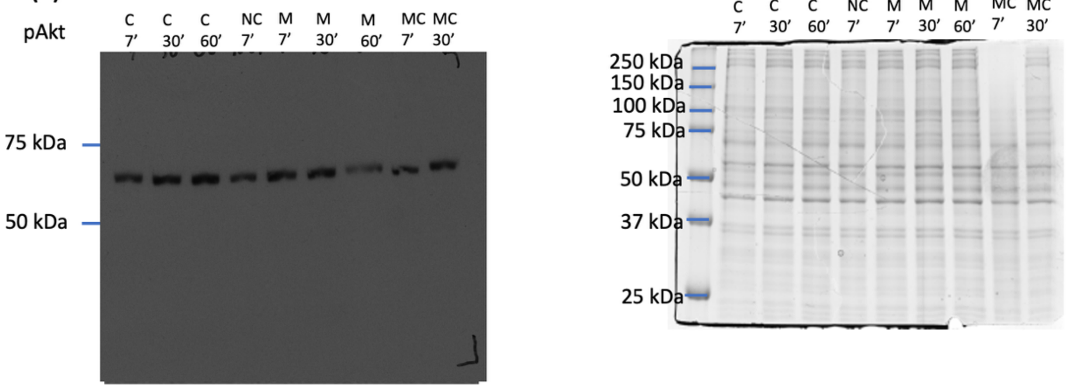
pAkt
C 7' C 30' C 60' NC 7' M 7' M 30' M 60' MC 7' MC 30'
75 kDa
50 kDa
C 7' C 30' C 60' NC 7' M 7' M 30' M 60' MC 7' MC 30'
250 kDa
150 kDa
100 kDa
75 kDa
50 kDa
37 kDa
25 kDa

(I)

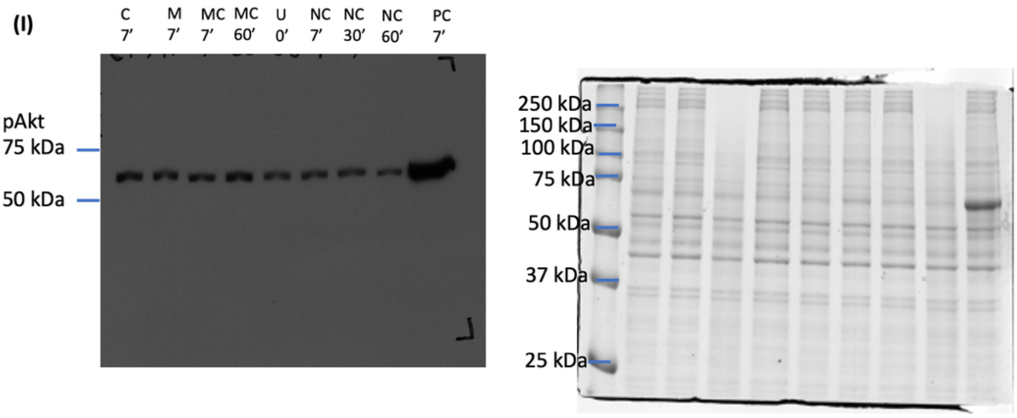
C 7′
M 7′
MC 7′
MC 60′
U 0′
NC 7′
NC 30′
NC 60′
PC 7′
pAkt
75 kDa
50 kDa
250 kDa
150 kDa
100 kDa
75 kDa
50 kDa
37 kDa
25 kDa

(J)

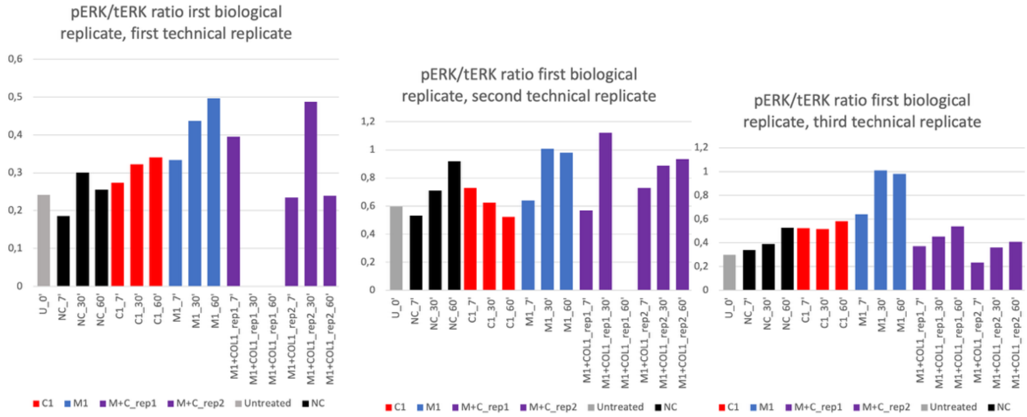
pERK/tERK ratio irst biological replicate, first technical replicate
pERK/tERK ratio first biological replicate, second technical replicate
pERK/tERK ratio first biological replicate, third technical replicate
C1
M1
M+C_rep1
M+C_rep2
Untreated
NC

(K)

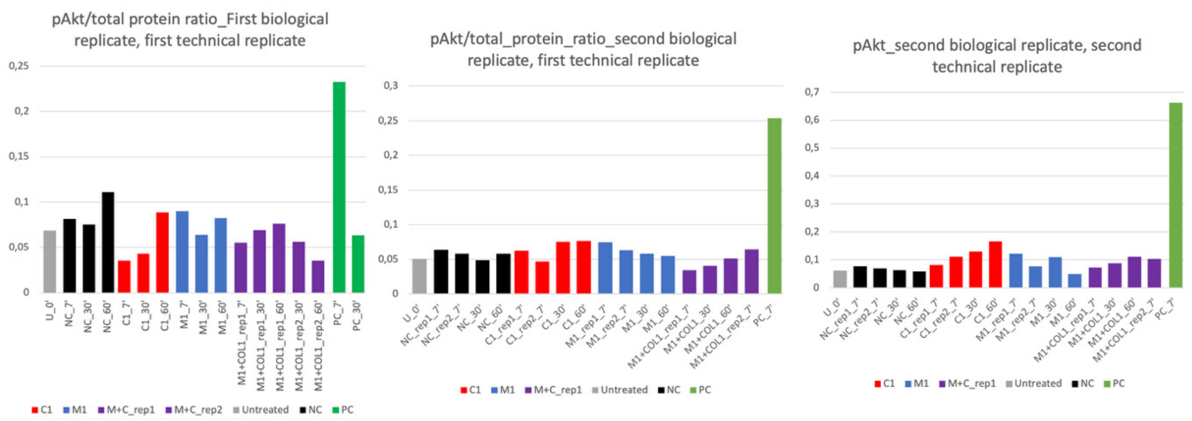

Supplementary Figure S3: A-C: individual images of western blots for pERK (top row) and total ERK (bottom row). Matrikine + collagen type I treated samples on both membranes are technical replicates from each other. Lanes indicated by an arrow were not considered due to stains. D-I: individual images of western blots for pAkt and analytical gels. Panels D-E, F-G, and H-I were run together, respectively. Panels D-E: Matrikine + collagen type I treated samples on both membranes are technical replicates from each other. Lanes indicated by an arrow were not considered due to stains. Panels F-I: samples obtained at time point 7 were run in duplicate to assess technical reproducibility. Panel J shows the phosphorylated ERK over total ERK ratio for all 3 replicates; while a decrease in pERK for matrikine treated samples at time point 7 could be observed, which gradually recovers, the effect size is small and the reproducibility across the technical replicates poor. Panel K shows the phosphorylated Akt over total protein quantification (using Coomassie on mirrogels) is shown. While an increase in pAkt ratio over time can be observed for the collagen treated samples, the change is subtle and poorly reproducible. Abbreviations: C = collagen type I treated samples, M = matrikine 1-treated samples, MC = matrikine 1 + collagen type I-treated samples, NC = negative control (acetic acid dilution used to solubilize collagen), PC = positive control (5 v/v% FBS + 1mM hydrogen peroxide), and U = untreated (without any compounds added) cells. Numbers denote the different time point (e.g. 7 min, 30 min, 60 min) the samples were collected at.

(A)

❌ 60S ribosomal protein L31

#1	b^{+}	b^{2+}	Seq.	y^{+}	y^{2+}
1	88.03930	44.52329	S		
2	159.07642	80.04185	A	1979.99502	990.50115
3	272.16048	136.58388	I	1908.95790	954.98259
4	386.20341	193.60534	N	1795.87384	898.44056
5	515.24600	258.12664	E	1681.83091	841.41909
6	614.31442	307.66085	V	1552.78832	776.89780
7	713.38283	357.19505	V	1453.71991	727.36359
8	814.43051	407.71889	T	1354.65149	677.82938
9	970.53162	485.76945	R	1253.60381	627.30554
10	1099.57421	550.29074	E	1097.50270	549.25499
11	1262.63754	631.82241	Y	968.46011	484.73369
12	1443.65155	722.32941	T-Phospho	805.39678	403.20203
13	1556.73561	778.87145	I	624.38277	312.69502
14	1670.77854	835.89291	N	511.29871	256.15299
15	1783.86261	892.43494	I	397.25578	199.13153
16	1920.92152	960.96440	H	284.17172	142.58950
17			K	147.11280	74.06004

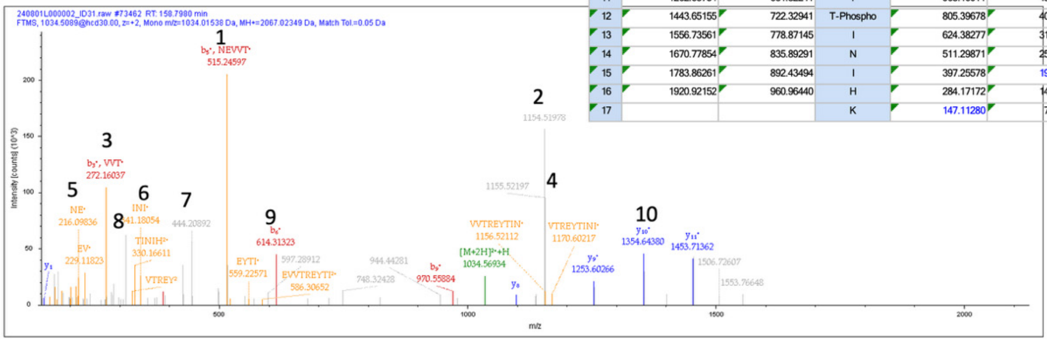

(B)

Actin alpha cardiac muscle (I)

#1	b⁺	b²⁺	b³⁺	Seq.	y⁺	y²⁺	y³⁺
1	116.03422	58.52075	39.34959	D			
2	229.11828	115.06278	77.04428	L	2208.99951	1105.00339	737.004
3	392.18161	196.59444	131.39872	Y	2095.91545	1048.46136	699.310
4	463.21873	232.11300	155.07776	A	1932.85212	966.92970	644.955
5	577.26165	289.13446	193.09207	N	1861.81500	931.41114	621.276
6	691.30458	346.15593	231.10638	N	1747.77208	874.38968	583.262
7	790.37299	395.69014	264.12918	V	1633.72915	817.36821	545.247
8	903.45706	452.23217	301.82387	L	1534.66074	767.83401	512.225
9	1070.45542	535.73135	357.48999	S-Phospho	1421.57667	711.29197	474.530
10	1127.47688	564.24208	376.49714	G	1254.57831	627.79279	418.864
11	1184.49835	592.75281	395.50430	G	1197.55685	599.28206	399.857
12	1285.54602	643.27665	429.18686	T	1140.53539	570.77133	380.849
13	1386.59370	693.80049	462.86942	T	1039.48771	520.24749	347.167
14	1533.62910	767.31819	511.88122	M-Oxidation	938.44003	469.72365	313.484
15	1696.69243	848.84985	566.23566	Y	791.40463	396.20595	264.473
16	1793.74519	897.37624	598.58658	P	628.34130	314.67429	210.118
17	1850.76666	925.88697	617.59374	G	531.28854	266.14791	177.767
18	1963.85072	982.42900	655.28842	I	474.26707	237.63717	158.760
19	2034.88784	1017.94756	678.96746	A	361.18301	181.09514	121.065
20	2149.91478	1075.46103	717.30978	D	290.14590	145.57659	97.386
21				R	175.11895	88.06311	59.044

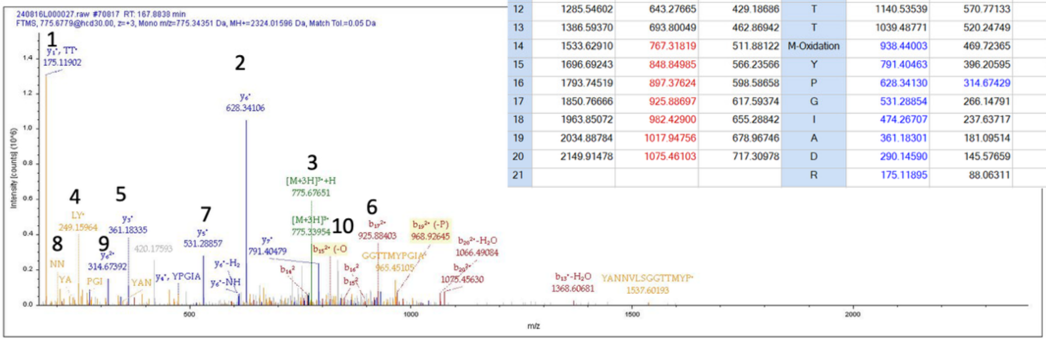

(C)

Actin alpha cardiac muscle (II)

#1	b⁺	b²⁺	b³⁺	Seq.	y⁺	y²⁺	y³⁺
1	116.03422	58.52075	39.34959	D			
2	229.11828	115.06278	77.04428	L	2193.00460	1097.00594	731.6
3	472.14794	236.57761	158.05417	Y-Phospho	2079.92053	1040.46390	693.9
4	543.18506	272.09617	181.73320	A	1836.89087	918.94907	612.9
5	657.22798	329.11763	219.74751	N	1765.85376	883.43052	589.2
6	771.27091	386.13909	257.76182	N	1651.81083	826.40905	551.2
7	870.33933	435.67330	290.78463	V	1537.76790	769.38759	513.2
8	983.42339	492.21533	328.47931	L	1438.69949	719.85338	480.2
9	1070.45542	535.73135	357.48999	S	1325.61543	663.31135	442.5
10	1127.47688	564.24208	376.49714	G	1238.58340	619.79534	413.5
11	1184.49835	592.75281	395.50430	G	1181.56193	591.28461	394.5
12	1285.54602	643.27665	429.18686	T	1124.54047	562.77387	375.5
13	1386.59370	693.80049	462.86942	T	1023.49279	512.25003	341.8
14	1517.63419	759.32073	506.54958	M	922.44511	461.72620	308.1
15	1680.69751	840.85240	560.90402	Y	791.40463	396.20595	264.4
16	1777.75028	889.37878	593.25494	P	628.34130	314.67429	210.1
17	1834.77174	917.88951	612.26210	G	531.28854	266.14791	177.7
18	1947.85581	974.43154	649.95679	I	474.26707	237.63717	158.7
19	2018.89292	1009.95010	673.63582	A	361.18301	181.09514	121.0
20	2133.91986	1067.46357	711.97814	D	290.14590	145.57659	97.3
21				R	175.11895	88.06311	59.0

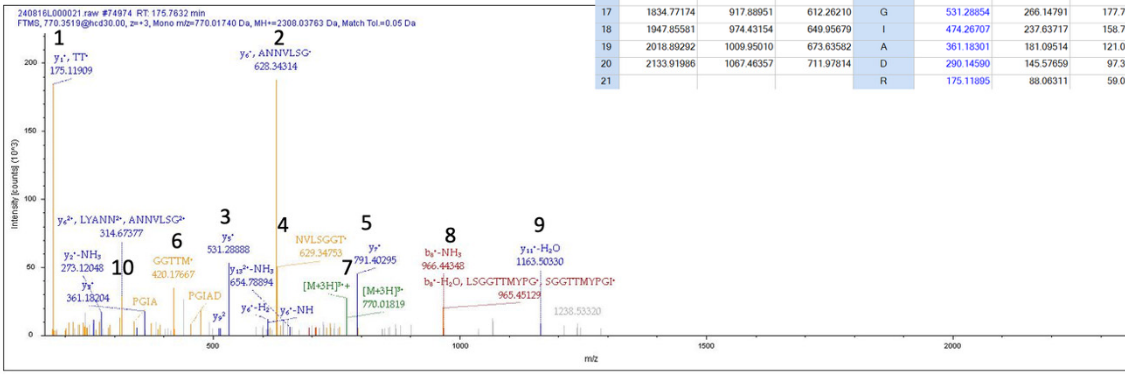

(D)

Adenylyl cyclase-associated protein 1

#1	b⁺	b²⁺	b³⁺	Seq.	y⁺	y²⁺	y³⁺
1	88.03930	44.52329	30.01795	S			
2	145.06077	73.03402	49.02511	G	1830.91498	915.96113	610.976
3	242.11353	121.56040	81.37603	P	1773.89351	887.45039	591.969
4	370.20850	185.60789	124.07435	K	1676.84075	838.92401	559.618
5	467.26126	234.13427	156.42527	P	1548.74579	774.87653	516.920
6	614.32967	307.66847	205.44808	F	1451.69302	726.35015	484.569
7	701.36170	351.18449	234.45875	S	1304.62461	652.81594	435.546
8	772.39882	386.70305	258.13779	A	1217.59258	609.29993	406.535
9	869.45158	435.22943	290.48871	P	1146.55547	573.78137	382.856
10	997.54654	499.27691	333.18703	K	1049.50270	525.25499	350.505
11	1094.59931	547.80329	365.53795	P	921.40774	461.20751	307.807
12	1222.65788	611.83258	408.22415	Q	824.35498	412.68113	275.456
13	1323.70556	662.35642	441.90670	T	696.29640	348.65184	232.770
14	1490.70392	745.85560	497.57282	S-Phospho	595.24872	298.12800	199.087
15	1587.75668	794.38198	529.92375	P	428.25036	214.62882	143.421
16	1674.78871	837.89799	558.93442	S	331.19760	166.10244	111.070
17	1771.84148	886.42438	591.28534	P	244.16557	122.58642	82.060
18				K	147.11280	74.06004	49.709

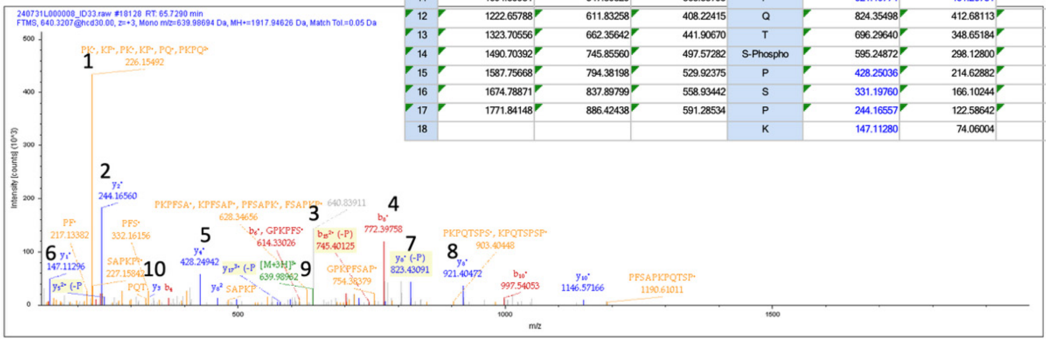

(E)

ATPase family AAA domain-containing protein 3A

#1	b⁺	b²⁺	Seq.	y⁺	y²⁺	#2
1	114.09134	57.54931	I			8
2	295.10535	148.05631	T-Phospho	881.44921	441.22824	7
3	394.17376	197.59052	V	700.43520	350.72124	6
4	507.25783	254.13255	L	601.36679	301.18703	5
5	636.30042	318.65385	E	488.28272	244.64500	4
6	707.33753	354.17241	A	359.24013	180.12370	3
7	820.42160	410.71444	L	288.20302	144.60515	2
8			R	175.11895	88.06311	1

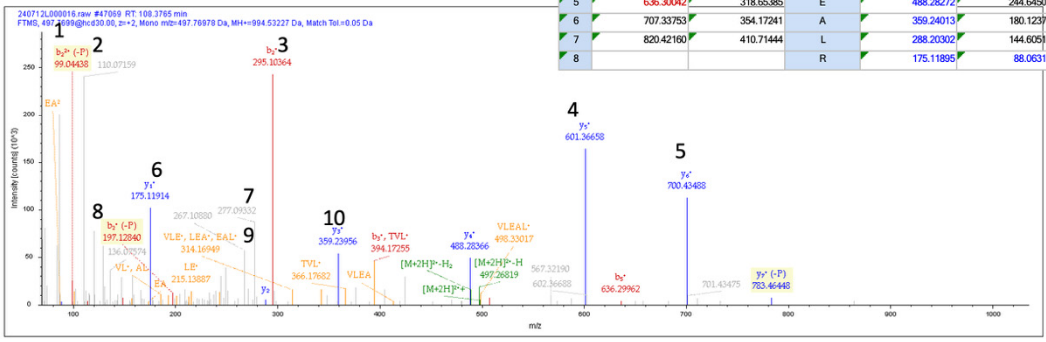

(F)

Beta-actin-like protein 2

#1	b⁺	b²⁺	b³⁺	Seq.	y⁺	y²⁺	y³⁺
1	116.03422	58.52075	39.34959	D			
2	229.11828	115.06278	77.04428	L	2165.99370	1083.50049	722.669
3	392.18161	196.59444	131.39872	Y	2052.90963	1026.95845	684.974
4	463.21873	232.11300	155.07776	A	1889.84630	945.42679	630.620
5	577.26165	289.13446	193.09207	N	1818.80919	909.90823	606.941
6	678.30933	339.65830	226.77463	T	1704.76626	852.88677	568.926
7	777.37775	389.19251	259.79743	V	1603.71858	802.36293	535.244
8	890.46181	445.73454	297.49212	L	1504.65017	752.82872	502.221
9	977.49384	489.25056	326.50280	S	1391.56611	696.28669	464.526
10	1034.51530	517.76129	345.50995	G	1304.53408	652.77068	435.516
11	1091.53677	546.27202	364.51711	G	1247.51261	624.25995	416.509
12	1258.53512	629.77120	420.18323	S-Phospho	1190.49115	595.74921	397.501
13	1359.58280	680.29504	453.86579	T	1023.49279	512.25003	341.835
14	1490.62329	745.81528	497.54595	M	922.44511	461.72620	308.153
15	1653.68662	827.34695	551.90039	Y	791.40463	396.20595	264.473
16	1750.73938	875.87333	584.25131	P	628.34130	314.67429	210.118
17	1807.76084	904.38406	603.25847	G	531.28854	266.14791	177.767
18	1920.84491	960.92609	640.95315	I	474.26707	237.63717	158.760
19	1991.88202	996.44465	664.63219	A	361.18301	181.09514	121.065
20	2106.90896	1053.95812	702.97451	D	290.14590	145.57659	97.386
21				R	175.11895	88.06311	59.044

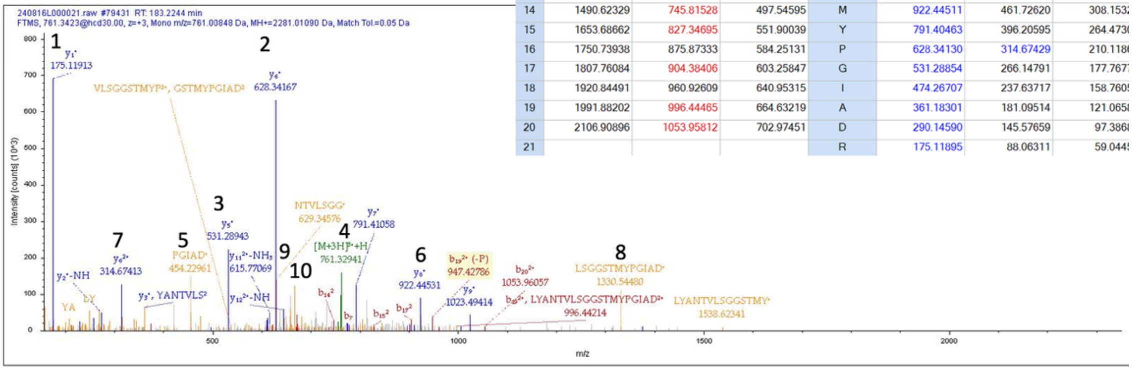

(G)

Calnexin

#1	b⁺	b²⁺	b³⁺	Seq.	y⁺	y²⁺	y³⁺
1	72.04439	36.52583	24.68631	A			
2	201.08698	101.04713	67.70051	E	1437.63696	719.32212	479.883
3	330.12958	165.56843	110.71471	E	1308.59437	654.80082	436.869
4	445.15652	223.08190	149.05702	D	1179.55178	590.27953	393.855
5	574.19911	287.60319	192.07122	E	1064.52483	532.76606	355.513
6	687.28318	344.14523	229.76591	I	935.48224	468.24476	312.498
7	800.36724	400.68726	267.46060	L	822.39818	411.70273	274.804
8	914.41017	457.70872	305.47491	N	709.31411	355.16070	237.109
9	1070.51128	535.75928	357.50861	R	595.27119	298.13923	199.095
10	1237.50964	619.25846	413.17473	S-Phospho	439.17008	220.08868	147.061
11	1334.56240	667.78484	445.52565	P	272.17172	136.58950	91.395
12				R	175.11895	88.06311	59.044

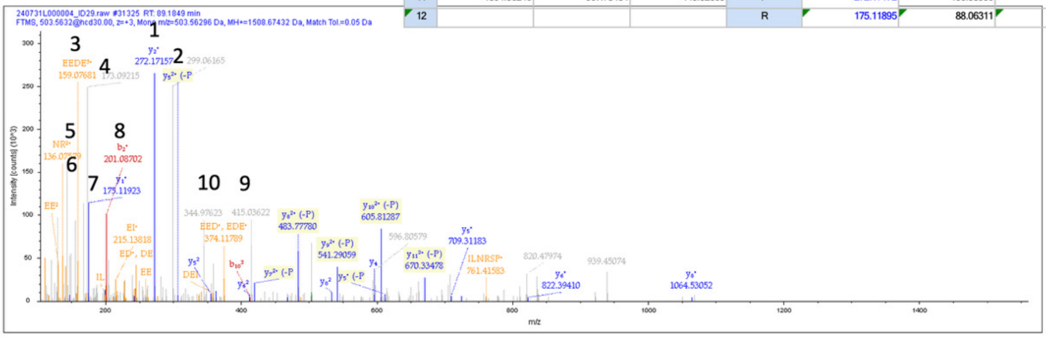

(H)

Calumenin

#1	b⁺	b²⁺	b³⁺	Seq.	y⁺	y²⁺	y³⁺
1	100.07569	50.54148	34.03008	V			
2	237.13460	119.07094	79.71639	H	2459.97759	1230.49243	820.664
3	351.17753	176.09240	117.73069	N	2322.91868	1161.96298	774.977
4	466.20447	233.60587	156.07301	D	2208.87575	1104.94151	736.963
5	537.24159	269.12443	179.75205	A	2093.84881	1047.42804	698.621
6	665.30016	333.15372	222.43824	Q	2022.81169	1011.90948	674.942
7	832.29852	416.65290	278.10436	S-Phospho	1894.75312	947.88020	632.255
8	979.36694	490.18711	327.12716	F	1727.75476	864.38102	576.589
9	1094.39388	547.70058	365.46948	D	1580.68634	790.84681	527.566
10	1257.45721	629.23224	419.82392	Y	1465.65940	733.33334	489.224
11	1372.48415	686.74571	458.16623	D	1302.59607	651.80167	434.870
12	1509.54306	755.27517	503.85254	H	1187.56913	594.28820	396.527
13	1624.57001	812.78864	542.19485	D	1050.51022	525.75875	350.841
14	1695.60712	848.30720	565.87389	A	935.48327	468.24527	312.499
15	1842.67553	921.84141	614.89670	F	864.44616	432.72672	288.820
16	1955.75960	978.38344	652.59138	L	717.37775	359.19251	239.797
17	2012.78106	1006.89417	671.59854	G	604.29368	302.65048	202.102
18	2083.81818	1042.41273	695.27758	A	547.27222	274.13975	183.095
19	2212.86077	1106.93402	738.29177	E	476.23510	238.62119	159.416
20	2341.90336	1171.45532	781.30597	E	347.19251	174.09989	116.402
21	2412.94047	1206.97388	804.98501	A	218.14992	109.57860	73.388
22				K	147.11280	74.06004	49.709

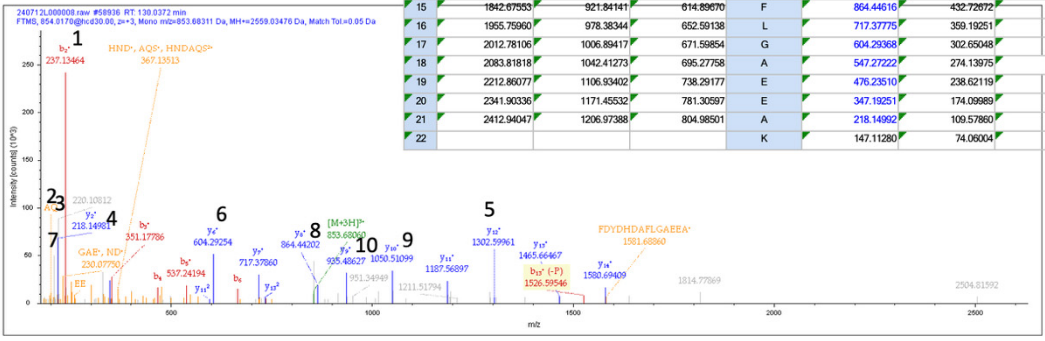

(I)

Drebrin

#1	b⁺	b²⁺	Seq.	y⁺	y²⁺	#2
1	114.09134	57.54931	L			8
2	201.12337	101.06532	S	875.41349	438.21039	7
3	368.12173	184.56450	S-Phospho	788.38147	394.69437	6
4	465.17449	233.09088	P	621.38311	311.19519	5
5	564.24291	282.62509	V	524.33034	262.66881	4
6	677.32697	339.16712	L	425.26193	213.13460	3
7	814.38588	407.69658	H	312.17786	156.59257	2
8			R	175.11895	88.06311	1

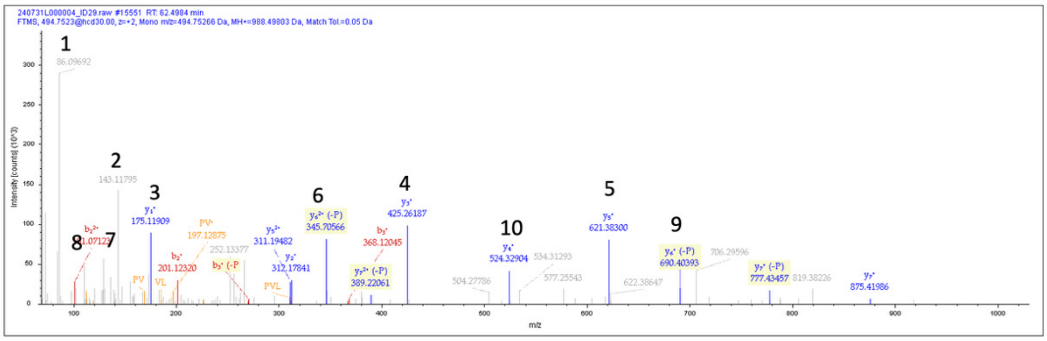

(J)

Endogenous retrovirus group K member 19 Env polyprotein

#1	b⁺	b²⁺	b³⁺	Seq.	y⁺	y²⁺	y³⁺
1	114.09134	57.54931	38.70196	L			
2	295.10535	148.05631	99.03997	T-Phospho	1080.43874	540.72301	360.81776
3	394.17376	197.59052	132.06277	V	899.42473	450.21600	300.47976
4	465.21088	233.10908	155.74181	A	800.35631	400.68179	267.45696
5	632.20924	316.60826	211.40793	S-Phospho	729.31920	365.16324	243.77792
6	769.26815	385.13771	257.09423	H	562.32084	281.66406	188.11180
7	906.32706	453.66717	302.78054	H	425.26193	213.13460	142.42549
8	1019.41112	510.20920	340.47523	I	288.20302	144.60515	96.73919
9				R	175.11895	88.06311	59.04450

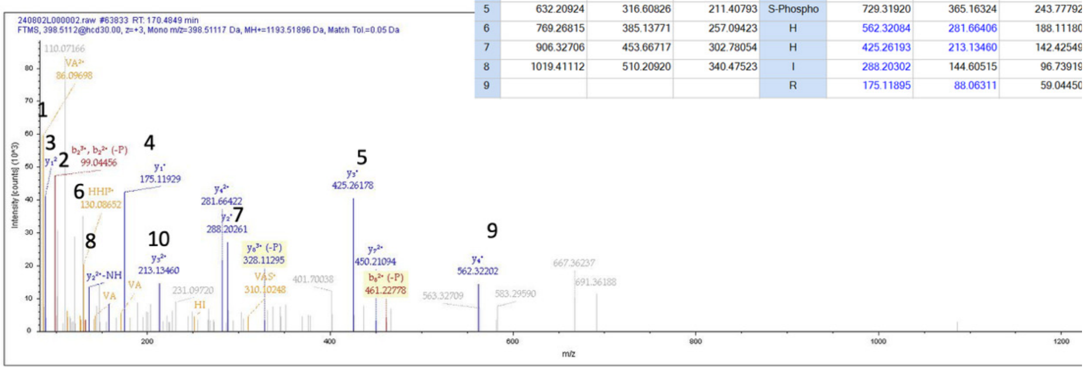

(K)

Eukaryotic translation initiation factor 3 subunit E

#1	b⁺	b²⁺	Seq.	y⁺	y²⁺
1	130.04987	65.52857	E		
2	243.13393	122.07061	I	1414.69777	707.852
3	486.16359	243.58543	Y-Phospho	1301.61371	651.310
4	600.20652	300.60690	N	1058.58405	529.795
5	729.24911	365.12820	E	944.54112	472.774
6	857.34408	429.17568	K	815.49853	408.252
7	986.38667	493.69697	E	687.40357	344.205
8	1099.47073	550.23900	L	558.36097	279.684
9	1212.55480	606.78104	L	445.27691	223.142
10	1340.61337	670.81033	Q	332.19285	166.600
11	1397.63484	699.32106	G	204.13427	102.570
12			K	147.11280	74.060

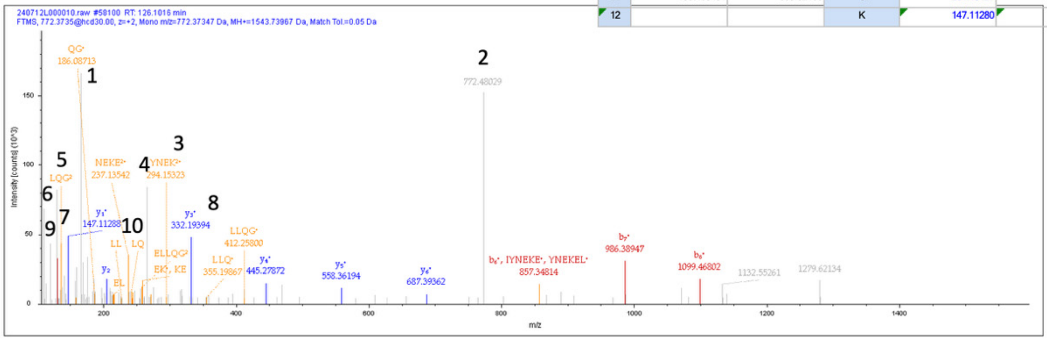

(L)

Fibrinogen gamma chain

#1	b^{+}	b^{2+}	Seq.	y^{+}	y^{2+}
1	129.06585	65.03657	Q		
2	216.09788	108.55258	S	1245.66429	623.335
3	273.11935	137.06331	G	1158.63226	579.819
4	386.20341	193.60534	L	1101.61080	551.309
5	629.23307	315.12017	Y-Phospho	988.52673	494.767
6	776.30148	388.65438	F	745.49707	373.252
7	889.38555	445.19641	I	598.42866	299.717
8	1017.48051	509.24389	K	485.34459	243.175
9	1114.53327	557.77028	P	357.24963	179.128
10	1227.61734	614.31231	L	260.19687	130.602
11			K	147.11280	74.060

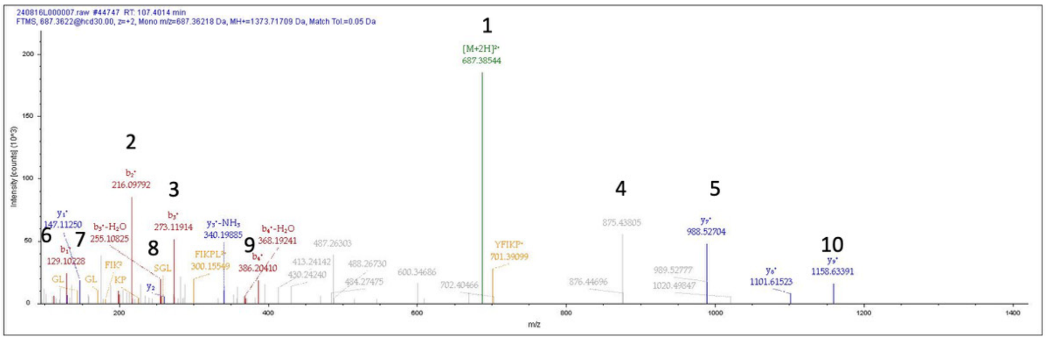

(M)

Heat shock protein beta-1

#1	b^{+}	b^{2+}	Seq.	y^{+}	y^{2+}
1	129.06585	65.03657	Q		
2	242.14992	121.57860	L	1027.48197	514.244
3	409.14828	205.07778	S-Phospho	914.39790	457.702
4	496.18031	248.59379	S	747.39954	374.203
5	553.20177	277.10452	G	660.36752	330.687
6	652.27018	326.63873	V	603.34605	302.176
7	739.30221	370.15474	S	504.27764	252.642
8	868.34480	434.67604	E	417.24561	209.126
9	981.42887	491.21807	I	288.20302	144.605
10			R	175.11895	88.063

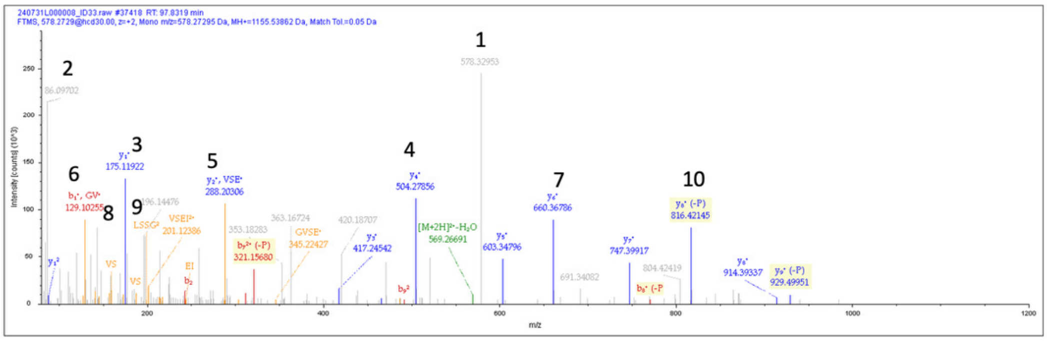

(N) Heat shock protein HSP 90-alpha

#1	b⁺	b²⁺	b³⁺	Seq.	y⁺	y²⁺	y³⁺
1	130.04987	65.52857	44.02147	E			
2	217.08190	109.04459	73.03215	S	2271.93880	1136.47304	757.984
3	346.12449	173.56588	116.04635	E	2184.90677	1092.95703	728.973
4	461.15143	231.07936	154.38866	D	2055.86418	1028.43573	685.959
5	589.24640	295.12684	197.08698	K	1940.83724	970.92226	647.617
6	686.29916	343.65322	229.43790	P	1812.74228	906.87478	604.918
7	815.34175	408.17452	272.45210	E	1715.68951	858.34839	572.568
8	928.42582	464.71655	310.14679	I	1586.64692	793.82710	529.553
9	1057.46841	529.23784	353.16099	E	1473.56286	737.28507	491.859
10	1172.49535	586.75132	391.50330	D	1344.52026	672.76377	448.844
11	1271.56377	636.28552	424.52611	V	1229.49332	615.25030	410.502
12	1328.58523	664.79625	443.53326	G	1130.42491	565.71609	377.479
13	1495.58359	748.29543	499.19938	S-Phospho	1073.40344	537.20536	358.472
14	1610.61053	805.80891	537.54170	D	906.40508	453.70618	302.806
15	1739.65313	870.33020	580.55589	E	791.37814	396.19271	264.464
16	1868.69572	934.85150	623.57009	E	662.33555	331.67141	221.450
17	1997.73831	999.37279	666.58429	E	533.29295	267.15011	178.435
18	2126.78091	1063.89409	709.59849	E	404.25036	202.62882	135.421
19	2254.87587	1127.94157	752.29681	K	275.20777	138.10752	92.407
20				K	147.11280	74.06004	49.709

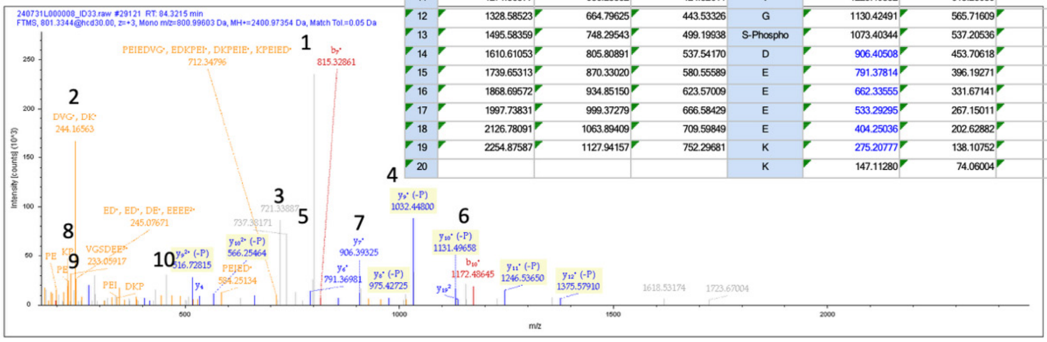

(O) Heat shock protein HSP 90-beta (I)

#1	b⁺	b²⁺	b³⁺	Seq.	y⁺	y²⁺	y³⁺
1	130.04987	65.52857	44.02147	E			
2	258.14483	129.57605	86.71980	K	1815.75317	908.38023	605.922
3	387.18743	194.09735	129.73399	E	1687.65821	844.33274	563.224
4	500.27149	250.63938	167.42868	I	1558.61562	779.81145	520.210
5	667.26985	334.13856	223.09480	S-Phospho	1445.53155	723.26942	482.515
6	782.29679	391.65203	261.43712	D	1278.53320	639.77024	426.849
7	897.32373	449.16551	299.77943	D	1163.50625	582.25676	388.506
8	1026.36633	513.68680	342.79363	E	1048.47931	524.74329	350.164
9	1097.40344	549.20536	366.47266	A	919.43672	460.22200	307.150
10	1226.44603	613.72666	409.48686	E	848.39960	424.70344	283.471
11	1355.48863	678.24795	452.50106	E	719.35701	360.18214	240.457
12	1484.53122	742.76925	495.51526	E	590.31442	295.66085	197.442
13	1612.62618	806.81673	538.21358	K	461.27182	231.13955	154.428
14	1669.64765	835.32746	557.22073	G	333.17686	167.09207	111.730
15	1798.69024	899.84876	600.23493	E	276.15540	138.58134	92.723
16				K	147.11280	74.06004	49.709

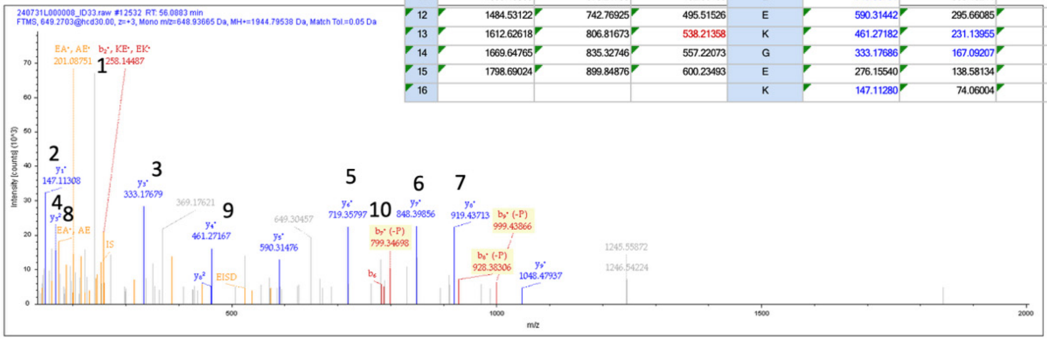

(P) Heat shock protein HSP 90-beta (II)

#1	b⁺	b²⁺	Seq.	y⁺	y²⁺
1	114.09134	57.54931	I		
2	243.13393	122.07061	E	1461.49008	731.248
3	358.16088	179.58408	D	1332.44749	666.727
4	457.22929	229.11828	V	1217.42055	609.213
5	514.25075	257.62902	G	1118.35213	559.679
6	681.24911	341.12820	S-Phospho	1061.33067	531.168
7	796.27606	398.64167	D	894.33231	447.669
8	925.31865	463.16296	E	779.30537	390.156
9	1054.36124	527.68426	E	650.26278	325.635
10	1169.38819	585.19773	D	521.22018	261.113
11	1284.41513	642.71120	D	406.19324	203.600
12	1371.44716	686.22722	S	291.16630	146.086
13	1428.46862	714.73795	G	204.13427	102.570
14			K	147.11280	74.060

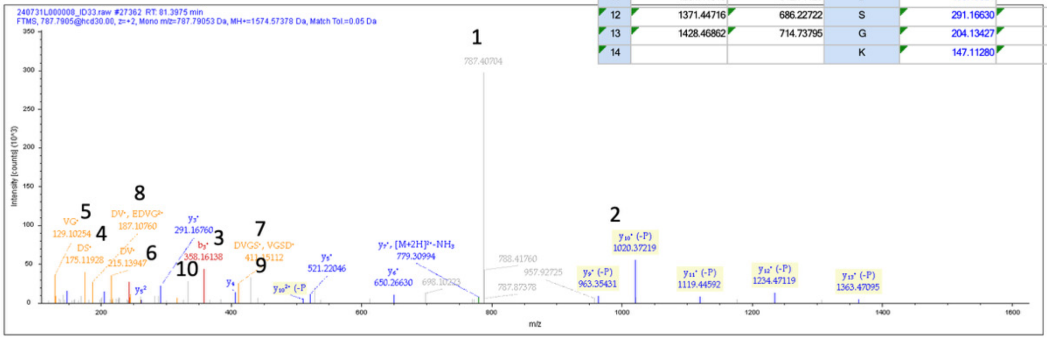

(Q) Heat shock protein HSP 90-beta (III)

#1	b⁺	b²⁺	Seq.	y⁺	y²⁺
1	114.09134	57.54931	I		
2	243.13393	122.07061	E	1704.61199	852.809
3	358.16088	179.58408	D	1575.56940	788.288
4	457.22929	229.11828	V	1460.54245	730.774
5	514.25075	257.62902	G	1361.47404	681.240
6	681.24911	341.12820	S-Phospho	1304.45258	652.729
7	796.27606	398.64167	D	1137.45422	569.230
8	925.31865	463.16296	E	1022.42727	511.717
9	1054.36124	527.68426	E	893.38468	447.195
10	1169.38819	585.19773	D	764.34209	382.674
11	1284.41513	642.71120	D	649.31515	325.161
12	1371.44716	686.22722	S	534.28820	267.647
13	1428.46862	714.73795	G	447.25617	224.131
14	1556.56358	778.78543	K	390.23471	195.620
15	1671.59053	836.29890	D	262.13975	131.573
16			K	147.11280	74.060

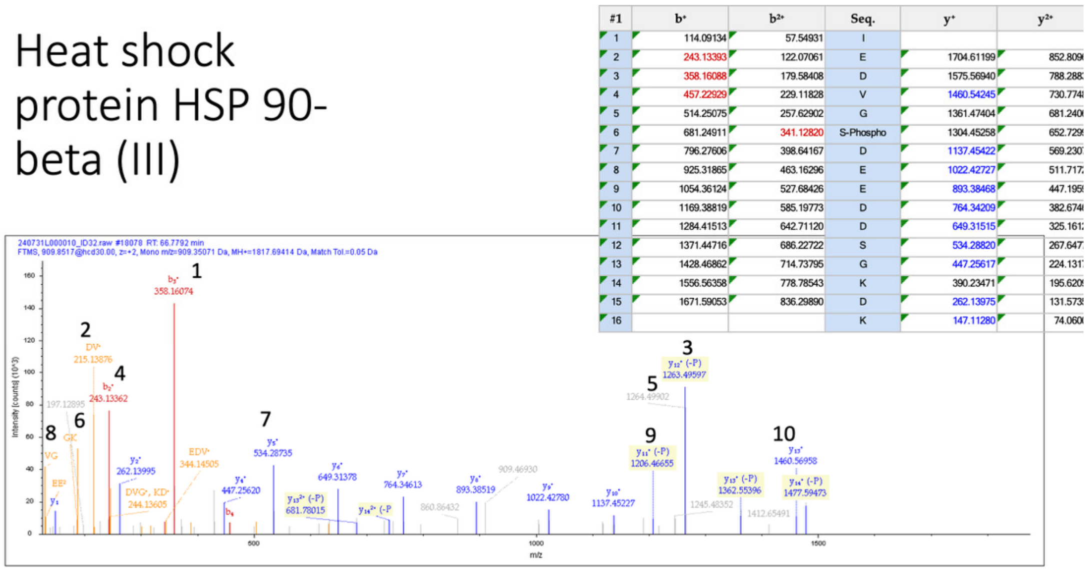

(R)

Heat shock protein HSP 90-beta (IV)

#1	b⁺	b²⁺	b³⁺	Seq.	y⁺	y²⁺	y³⁺
1	114.09134	57.54931	38.70196	I			
2	243.13393	122.07061	81.71616	E	1832.70695	916.85711	611.573
3	358.16088	179.58408	120.05848	D	1703.66436	852.33582	568.559
4	457.22929	229.11828	153.08128	V	1588.63742	794.82235	530.217
5	514.25075	257.62902	172.08844	G	1489.56900	745.28814	497.194
6	681.24911	341.12820	227.75456	S-Phospho	1432.54754	716.77741	478.187
7	796.27606	398.64167	266.09687	D	1265.54918	633.27823	422.521
8	925.31865	463.16296	309.11107	E	1150.52224	575.76476	384.178
9	1054.36124	527.68426	352.12527	E	1021.47964	511.24346	341.164
10	1169.38819	585.19773	390.46758	D	892.43705	446.72216	298.150
11	1284.41513	642.71120	428.80989	D	777.41011	389.20869	259.808
12	1371.44716	686.22722	457.82057	S	662.38317	331.69522	221.465
13	1428.46862	714.73795	476.82772	G	575.35114	288.17921	192.455
14	1556.56358	778.78543	519.52605	K	518.32967	259.66847	173.448
15	1671.59053	836.29890	557.86836	D	390.23471	195.62099	130.749
16	1799.68549	900.34638	600.56668	K	275.20777	138.10752	92.407
17				K	147.11280	74.06004	49.709

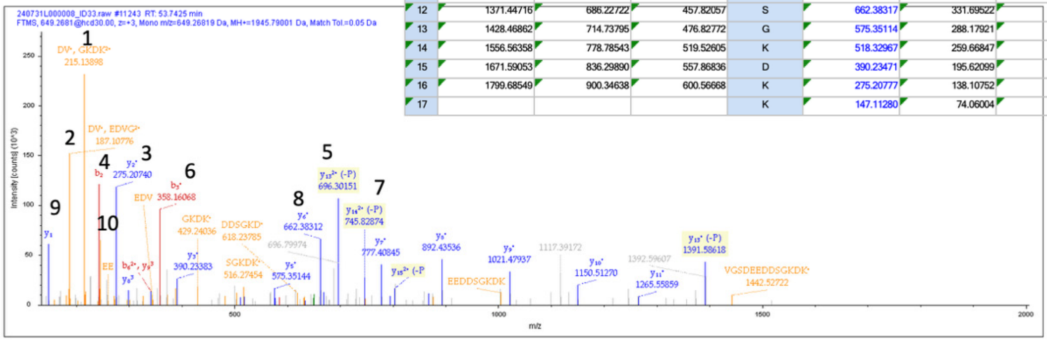

(S)

Heterogeneous nuclear ribonucleoprotein A1

#1	b⁺	b²⁺	Seq.	y⁺	y²⁺
1	88.03930	44.52329	S		
2	217.08190	109.04459	E	1292.58822	646.797
3	384.08026	192.54377	S-Phospho	1163.54563	582.276
4	481.13302	241.07015	P	996.54727	498.777
5	609.22798	305.11763	K	899.49451	450.250
6	738.27058	369.63893	E	771.39954	386.203
7	835.32334	418.16531	P	642.35695	321.682
8	964.36593	482.68661	E	545.30419	273.155
9	1092.42451	546.71589	Q	416.26159	208.634
10	1205.50858	603.25793	L	288.20302	144.605
11			R	175.11895	88.063

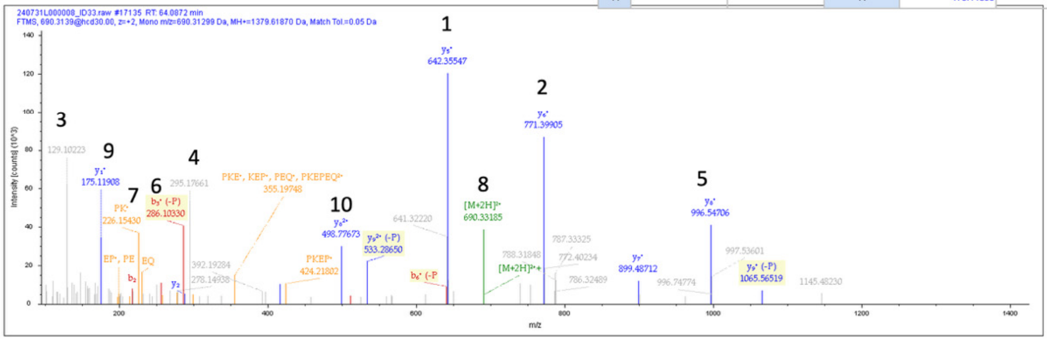

(T)

Histone H4

#1	b^+	b^{2+}	b^{3+}	Seq.	y^+	y^{2+}	y^{3+}
1	102.05496	51.53112	34.68984	T			
2	201.12337	101.06532	67.71264	V	1305.61602	653.31165	435.876
3	382.13738	191.57233	128.05064	T-Phospho	1206.54760	603.77744	402.854
4	453.17449	227.09088	151.72968	A	1025.53359	513.27044	342.516
5	600.20989	300.60858	200.74148	M-Oxidation	954.49648	477.75188	318.837
6	715.23683	358.12206	239.08380	D	807.46108	404.23418	269.825
7	814.30525	407.65626	272.10660	V	692.43414	346.72071	231.482
8	913.37366	457.19047	305.12941	V	593.36572	297.18650	198.460
9	1076.43699	538.72213	359.48385	Y	494.29731	247.65229	165.437
10	1147.47410	574.24069	383.16289	A	331.23398	166.12063	111.082
11	1260.55817	630.78272	420.85757	L	260.19687	130.60207	87.403
12				K	147.11280	74.06004	49.709

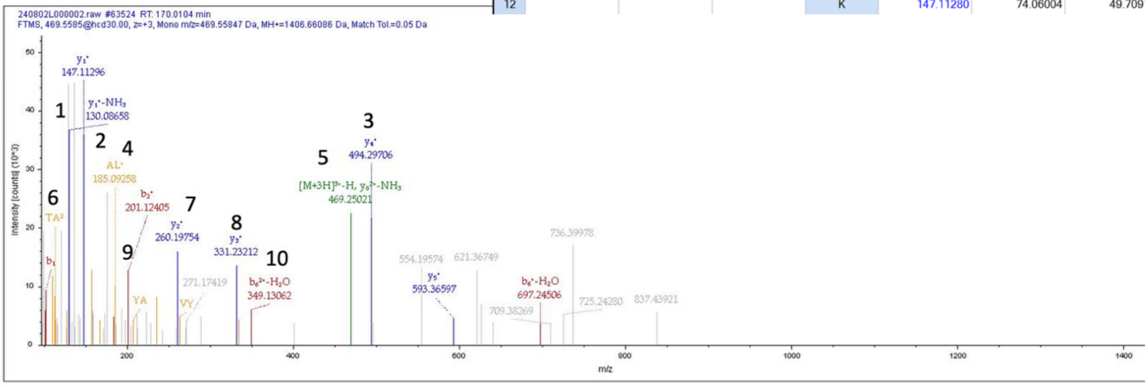

(U)

Myosin-13

#1	b^+	b^{2+}	Seq.	y^+	y^{2+}
1	129.10224	65.05476	K		
2	257.16082	129.08405	Q	1965.89134	983.449
3	328.19793	164.60260	A	1837.83276	919.420
4	443.22487	222.11608	D	1766.79565	883.901
5	610.22323	305.61525	S-Phospho	1651.76871	826.387
6	709.29165	355.14946	V	1484.77035	742.888
7	780.32876	390.66802	A	1385.70193	693.354
8	909.37135	455.18932	E	1314.66482	657.836
9	1022.45542	511.73135	L	1185.62223	593.314
10	1079.47688	540.24208	G	1072.53816	536.772
11	1208.51947	604.76338	E	1015.51670	508.261
12	1336.57805	668.79266	Q	886.47411	443.740
13	1449.66212	725.33470	I	758.41553	379.711
14	1564.68906	782.84817	D	645.33146	323.169
15	1678.73199	839.86963	N	530.30452	265.655
16	1791.81605	896.41166	L	416.26159	208.634
17	1919.87463	960.44095	Q	303.17753	152.092
18			R	175.11895	88.063

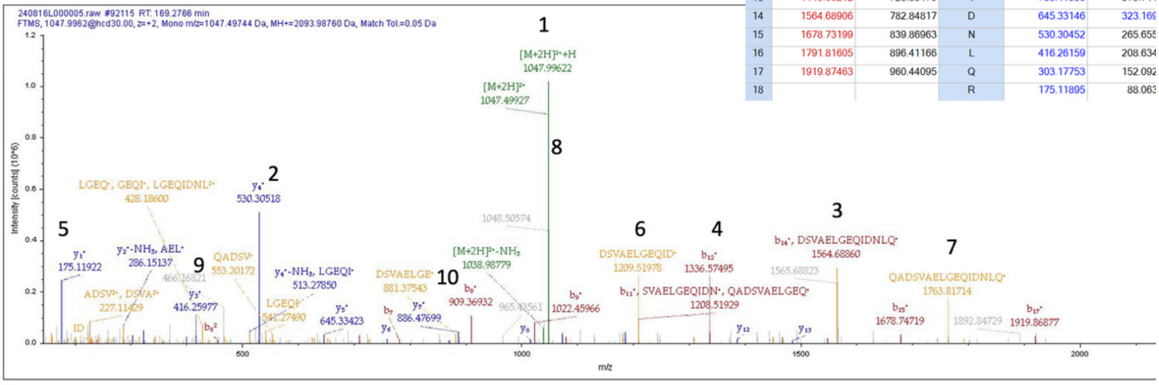

(V)

❌ Myosin 9

#1	b⁺	b²⁺	b³⁺	Seq.	y⁺	y²⁺	y³⁺
1	129.10224	65.05476	43.70560	K			
2	186.12370	93.56549	62.71275	G	2254.89834	1127.95281	752.304
3	257.16082	129.08405	86.39179	A	2197.87687	1099.44207	733.297
4	314.18228	157.59478	105.39894	G	2126.83976	1063.92352	709.618
5	429.20922	215.10825	143.74126	D	2069.81829	1035.41279	690.610
6	486.23069	243.61898	162.74841	G	1954.79135	977.89931	652.268
7	653.22905	327.11816	218.41453	S-Phospho	1897.76989	949.38858	633.261
8	768.25599	384.63163	256.75685	D	1730.77153	865.88940	577.595
9	897.29858	449.15293	299.77105	E	1615.74459	808.37593	539.253
10	1026.34118	513.67423	342.78524	E	1486.70199	743.85463	496.238
11	1125.40959	563.20843	375.80805	V	1357.65940	679.33334	453.224
12	1240.43653	620.72190	414.15036	D	1258.59099	629.79913	420.201
13	1297.45800	649.23264	433.15752	G	1143.56404	572.28566	381.859
14	1425.55296	713.28012	475.85584	K	1086.54258	543.77493	362.852
15	1496.59007	748.79867	499.53488	A	958.44762	479.72745	320.154
16	1611.61702	806.31215	537.87719	D	887.41050	444.20889	296.475
17	1668.63848	834.82288	556.88434	G	772.38356	386.69542	258.132
18	1739.67559	870.34143	580.56338	A	715.36210	358.18469	239.125
19	1868.71819	934.86273	623.57758	E	644.32498	322.66613	215.446
20	1939.75530	970.38129	647.25662	A	515.28239	258.14483	172.432
21	2067.85026	1034.42877	689.95494	K	444.24527	222.62628	148.753
22	2164.90303	1082.95515	722.30586	P	316.15031	158.57879	106.054
23	2235.94014	1118.47371	745.98490	A	219.09755	110.05241	73.704
24				E	148.06043	74.53386	50.025

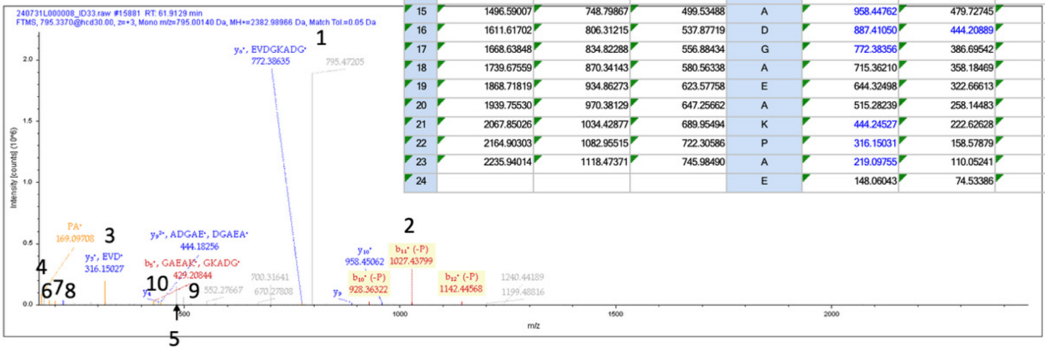

(W)

❌ Nestin

#1	b⁺	b²⁺	b³⁺	Seq.	y⁺	y²⁺	y³⁺
1	157.10839	79.05783	53.04098	R			
2	270.19245	135.59986	90.73567	L	3135.73567	1568.37147	1045.91
3	327.21392	164.11060	109.74282	G	3022.65160	1511.82944	1008.22
4	414.24594	207.62661	138.75350	S	2965.63014	1483.31871	989.21
5	527.33001	264.16864	176.44819	L	2878.59811	1439.80269	960.20
6	640.41407	320.71067	214.14287	L	2765.51405	1383.26066	922.50
7	737.46684	369.23706	246.49380	P	2652.42998	1326.71863	884.81
8	836.53525	418.77126	279.51660	V	2555.37722	1278.19225	852.46
9	949.61931	475.31329	317.21129	L	2456.30881	1228.65804	819.44
10	1116.61767	558.81247	372.87741	S-Phospho	2343.22474	1172.11601	781.74
11	1213.67044	607.33886	405.22833	P	2176.22638	1088.61683	726.08
12	1314.71811	657.86270	438.91089	T	2079.17362	1040.09045	693.72
13	1401.75014	701.37871	467.92157	S	1978.12594	989.56661	660.04
14	1514.83421	757.92074	505.61625	L	1891.09391	946.05059	631.03
15	1611.88697	806.44712	537.96717	P	1778.00985	889.50856	593.34
16	1698.91900	849.96314	566.97785	S	1680.95708	840.98218	560.99
17	1795.97176	898.48952	599.32877	P	1593.92506	797.46617	531.97
18	1909.05583	955.03155	637.02346	L	1496.87229	748.93978	499.62
19	2006.10859	1003.55793	669.37438	P	1383.78823	692.39775	461.93
20	2077.14570	1039.07649	693.05342	A	1286.73546	643.87137	429.58
21	2178.19338	1089.60033	726.73598	T	1215.69835	608.35281	405.90
22	2291.27745	1146.14236	764.43067	L	1114.65067	557.82897	372.22
23	2420.32004	1210.66366	807.44486	E	1001.56661	501.28694	334.52
24	2521.36772	1261.18750	841.12742	T	872.52402	436.76565	291.51
25	2618.42048	1309.71388	873.47835	P	771.47634	386.24181	257.83
26	2717.48890	1359.24809	906.50115	V	674.42357	337.71543	225.47
27	2814.54166	1407.77447	938.85207	P	575.35516	288.18122	192.45
28	2885.57877	1443.29302	962.53111	A	478.30240	239.65484	160.10
29	3032.64719	1516.82723	1011.55391	F	407.26528	204.13628	136.42
30	3145.73125	1573.36926	1049.24860	L	260.19687	130.60207	87.40
31				K	147.11280	74.06004	49.70

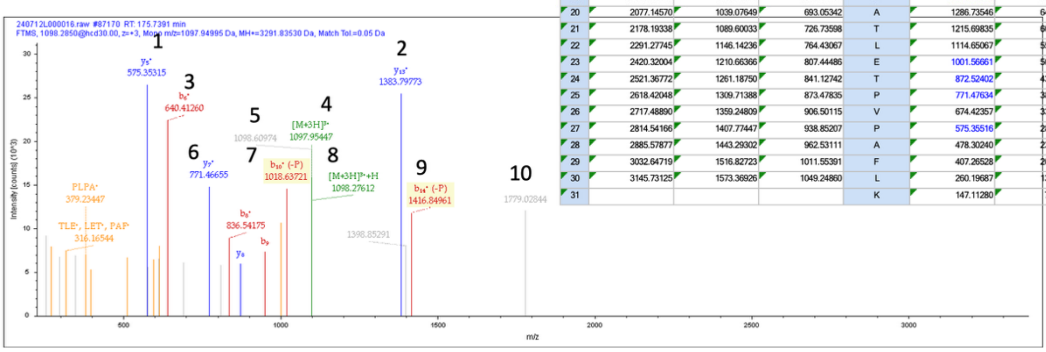

(X) Neuroblast differentiation associated protein AHNAK (I)

#1	b⁺	b²⁺	b³⁺	Seq.	y⁺	y²⁺	y³⁺
1	129.10224	65.05476	43.70560	K			
2	186.12370	93.56549	62.71275	G	1566.66966	783.83847	522.894
3	301.15065	151.07896	101.05507	D	1509.64820	755.32774	503.887
4	457.25176	229.12952	153.08877	R	1394.62125	697.81427	465.545
5	624.25012	312.62870	208.75489	S-Phospho	1238.52014	619.76371	413.511
6	721.30288	361.15508	241.10581	P	1071.52178	536.26453	357.845
7	850.34547	425.67637	284.12001	E	974.46902	487.73815	325.494
8	947.39824	474.20276	316.47093	P	845.42643	423.21685	282.480
9	1004.41970	502.71349	335.47808	G	748.37366	374.69047	250.129
10	1132.47828	566.74278	378.16428	Q	691.35220	346.17974	231.122
11	1233.52596	617.26662	411.84684	T	563.29362	282.15045	188.436
12	1419.60527	710.30627	473.87327	W	462.24594	231.62661	154.753
13	1520.65295	760.83011	507.55583	T	276.16663	138.58695	92.727
14				R	175.11895	88.06311	59.044

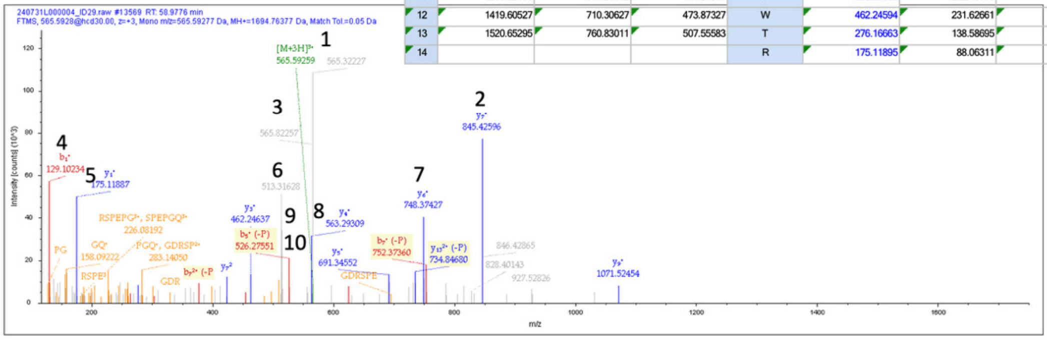

(Y) Neuroblast differentiation-associated protein AHNAK (II)

#1	b⁺	b²⁺	Seq.	y⁺	y²⁺
1	114.09134	57.54931	L		
2	211.14410	106.07569	P	1609.72176	805.364
3	298.17613	149.59170	S	1512.66899	756.838
4	355.19760	178.10244	G	1425.63696	713.322
5	442.22962	221.61845	S	1368.61550	684.811
6	499.25109	250.12918	G	1281.58347	641.295
7	570.28820	285.64774	A	1224.56201	612.784
8	641.32532	321.16630	A	1153.52489	577.266
9	808.32368	404.66548	S-Phospho	1082.48778	541.747
10	905.37644	453.19186	P	915.48942	458.248
11	1006.42412	503.71570	T	818.43666	409.721
12	1063.44558	532.22643	G	717.38898	359.198
13	1150.47761	575.74244	S	660.36752	330.687
14	1221.51472	611.26100	A	573.33549	287.171
15	1320.58314	660.79521	V	502.29837	251.652
16	1435.61008	718.30868	D	403.22996	202.118
17	1548.69414	774.85071	I	288.20302	144.605
18			R	175.11895	88.063

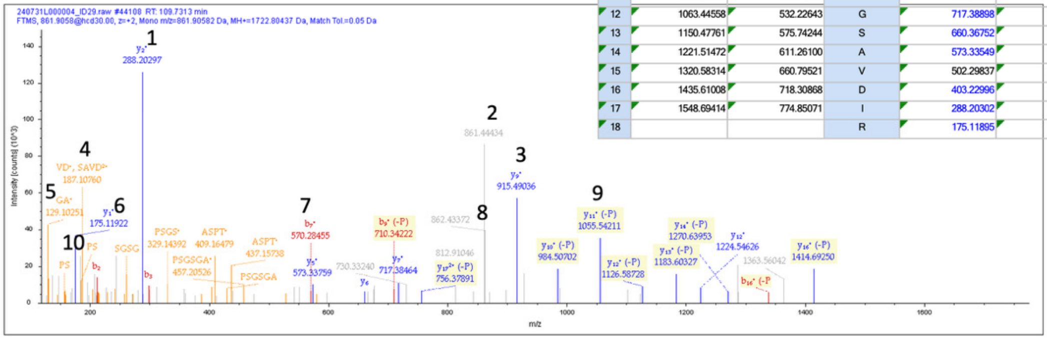

(Z)

❌ RAN GTPase

#1	b+	b2+	b3+	Seq.	y+	y2+	y3+
1	114.09134	57.54931	38.70196	L			
2	227.17540	114.09134	76.39665	L	2463.24906	1232.12817	821.754
3	383.27652	192.14190	128.43036	R	2350.16500	1175.58614	784.059
4	496.36058	248.68393	166.12504	L	2194.06388	1097.53558	732.026
5	553.38204	277.19466	185.13220	G	2080.97982	1040.99355	694.331
6	650.43481	325.72104	217.48312	P	2023.95836	1012.48282	675.324
7	778.52977	389.76852	260.18144	K	1926.90559	963.95643	642.973
8	945.52813	473.26770	315.84756	S-Phospho	1798.81063	899.90895	600.275
9	1032.56016	516.78372	344.85824	S	1631.81227	816.40977	544.608
10	1131.62857	566.31792	377.88104	V	1544.78024	772.89376	515.598
11	1244.71264	622.85996	415.57573	L	1445.71183	723.35955	482.575
12	1357.79670	679.40199	453.27042	I	1332.62776	666.81752	444.880
13	1428.83381	714.92054	476.94946	A	1219.54370	610.27549	407.186
14	1556.89239	778.94983	519.63565	Q	1148.50659	574.75693	383.507
15	1684.95097	842.97912	562.32184	Q	1020.44801	510.72764	340.820
16	1785.99865	893.50296	596.00440	T	892.38943	446.69835	298.134
17	1901.02559	951.01643	634.34671	D	791.34175	396.17452	264.452
18	2002.07327	1001.54027	668.02927	T	676.31481	338.66104	226.109
19	2089.10530	1045.05629	697.03995	S	575.26713	288.13720	192.427
20	2204.13224	1102.56976	735.38226	D	488.23510	244.62119	163.416
21	2301.18500	1151.09614	767.73319	P	373.20816	187.10772	125.074
22	2430.22760	1215.61744	810.74738	E	276.15540	138.58134	92.723
23				K	147.11280	74.06004	49.709

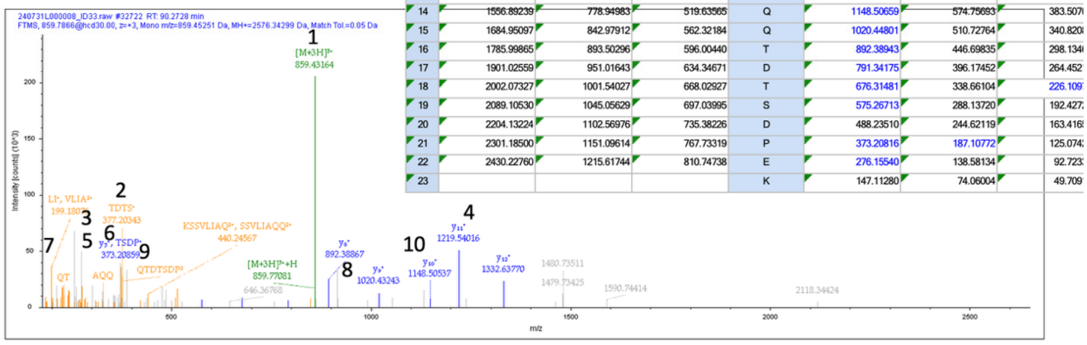

(AA)

RAS GTPase

#1	b+	b2+	Seq.	y+	y2+
1	88.03930	44.52329	S		
2	175.07133	88.03930	S	1877.86406	939.435
3	342.06969	171.53848	S-Phospho	1790.83203	895.919
4	439.12246	220.06487	P	1623.83368	812.420
5	510.15957	255.58342	A	1526.78091	763.894
6	607.21233	304.10981	P	1455.74380	728.375
7	678.24945	339.62836	A	1358.69103	679.849
8	793.27639	397.14183	D	1287.65392	644.330
9	906.36045	453.68387	I	1172.62698	586.817
10	977.39757	489.20242	A	1059.54291	530.275
11	1105.45615	553.23171	Q	988.50580	494.756
12	1206.50382	603.75555	T	860.44722	430.727
13	1305.57224	653.28976	V	759.39954	380.203
14	1433.63082	717.31905	Q	660.33113	330.669
15	1562.67341	781.84034	E	532.27255	266.639
16	1677.70035	839.35381	D	403.22996	202.118
17	1790.78442	895.89585	L	288.20302	144.605
18			R	175.11895	88.063

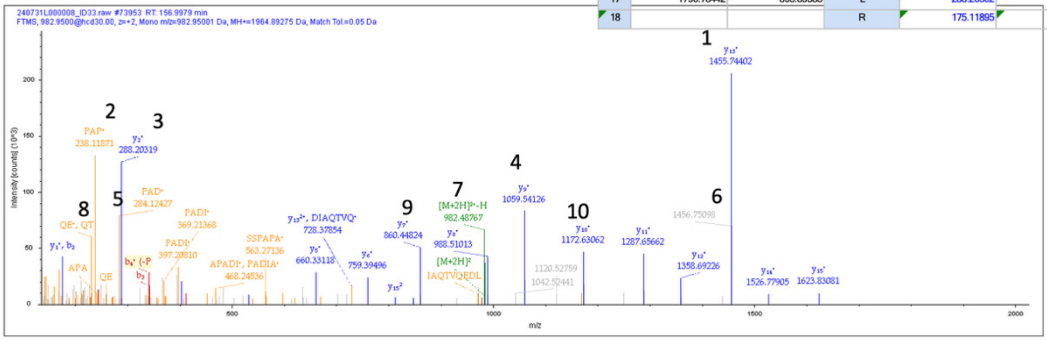

(AB)

Serine racemase

#1	b+	b2+	b3+	Seq.	y+	y2+	y3+
1	114.09134	57.54931	38.70196	L			
2	261.12674	131.06701	87.71376	M-Oxidation	1740.79142	870.89935	580.93
3	358.17950	179.59339	120.06469	P	1593.75602	797.38165	531.92
4	472.22243	236.61485	158.07899	N	1496.70325	748.85526	499.57
5	585.30650	293.15689	195.77368	L	1382.66032	691.83380	461.55
6	828.33615	414.67172	276.78357	Y-Phospho	1269.57626	635.29177	423.86
7	925.38892	463.19810	309.13449	P	1026.54660	513.77694	342.85
8	1022.44168	511.72448	341.48541	P	929.49384	465.25056	310.50
9	1151.48428	576.24578	384.49961	E	832.44107	416.72418	278.15
10	1252.53195	626.76962	418.18217	T	703.39848	352.20288	235.13
11	1365.61602	683.31165	455.87686	I	602.35080	301.67904	201.45
12	1436.65313	718.83020	479.55589	A	489.26674	245.13701	163.76
13	1551.68007	776.34368	517.89821	D	418.22962	209.61845	140.08
14	1608.70154	804.85441	536.90536	G	303.20268	152.10498	101.73
15	1707.76995	854.38861	569.92817	V	246.18122	123.59425	82.73
16				K	147.11280	74.06004	49.70

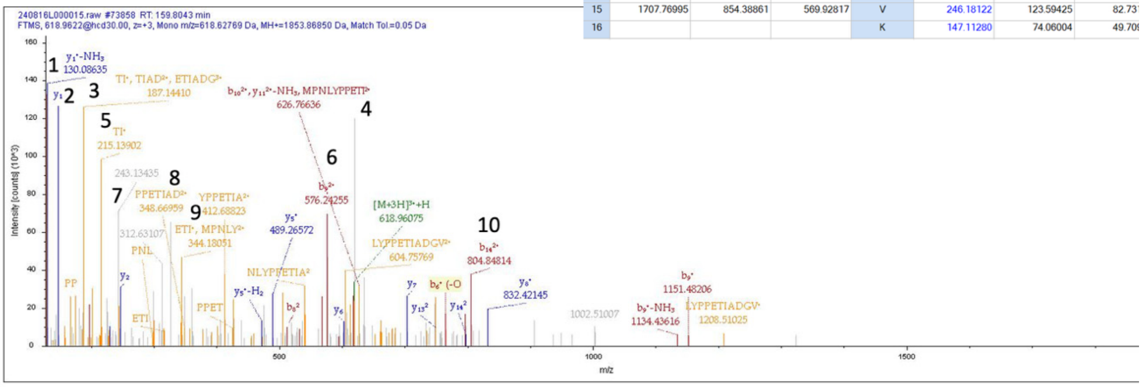

(AC)

Src substrate cortactin

#1	b+	b2+	Seq.	y+	y2+
1	114.09134	57.54931	L		
2	211.14410	106.07569	P	1477.62467	739.315
3	298.17613	149.59170	S	1380.57190	690.789
4	465.17449	233.09088	S-Phospho	1293.53988	647.273
5	562.22726	281.61727	P	1126.54152	563.774
6	661.29567	331.15147	V	1029.48875	515.248
7	824.35900	412.68314	Y	930.42034	465.713
8	953.40159	477.20443	E	767.35701	384.182
9	1068.42853	534.71791	D	638.31442	319.660
10	1139.46565	570.23646	A	523.28747	262.147
11	1210.50276	605.75502	A	452.25036	226.628
12	1297.53479	649.27103	S	381.21325	191.110
13	1444.60320	722.80524	F	294.18122	147.594
14			K	147.11280	74.060

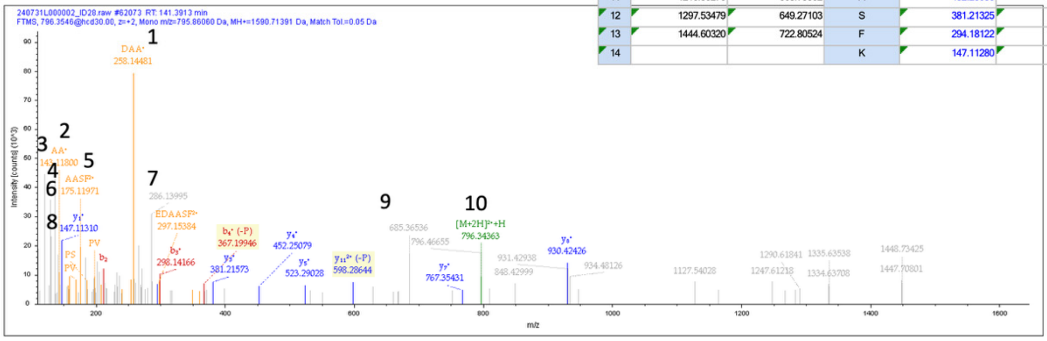

(AD)

Triosephosphate isomerase

#1	b⁺	b²⁺	b³⁺	Seq.	y⁺	y²⁺	y³⁺
1	100.07569	50.54148	34.03008	V			
2	197.12845	99.06787	66.38100	P	2172.96715	1086.98721	724.993
3	268.16557	134.58642	90.06004	A	2075.91438	1038.46083	692.642
4	383.19251	192.09989	128.40235	D	2004.87727	1002.94227	668.963
5	564.20652	282.60690	188.74036	T-Phospho	1889.85033	945.42880	630.621
6	693.24911	347.12820	231.75456	E	1708.83632	854.92180	570.283
7	792.31753	396.66240	264.77736	V	1579.79372	790.40050	527.269
8	891.38594	446.19661	297.80016	V	1480.72531	740.86629	494.246
9	1051.41659	526.21193	351.14371	Carbamidomet	1381.65690	691.33209	461.223
10	1122.45370	561.73049	374.82275	A	1221.62625	611.31676	407.880
11	1219.50647	610.25687	407.17367	P	1150.58914	575.79821	384.201
12	1316.55923	658.78325	439.52459	P	1053.53637	527.27182	351.850
13	1417.60691	709.30709	473.20715	T	956.48361	478.74544	319.499
14	1488.64402	744.82565	496.88619	A	855.43593	428.22160	285.816
15	1651.70735	826.35731	551.24063	Y	784.39882	392.70305	262.137
16	1764.79142	882.89935	588.93532	I	621.33549	311.17138	207.783
17	1879.81836	940.41282	627.27764	D	508.25142	254.62935	170.088
18	2026.88677	1013.94702	676.30044	F	393.22448	197.11588	131.746
19	2097.92389	1049.46558	699.97948	A	246.15607	123.58167	82.723
20				R	175.11895	88.06311	59.044

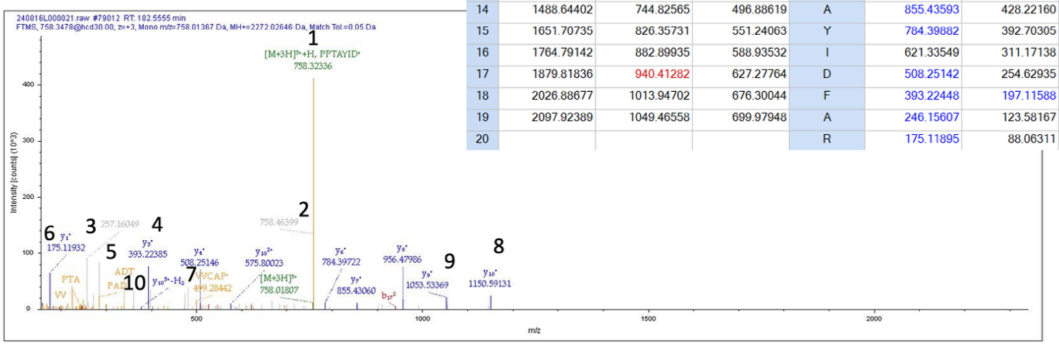

(AE)

Vimentin

#1	b⁺	b²⁺	Seq.	y⁺	y²⁺
1	88.03930	44.52329	S		
2	201.12337	101.06532	L	1421.64607	711.326
3	364.18670	182.59699	Y	1308.56201	654.784
4	435.22381	218.11554	A	1145.49868	573.252
5	522.25584	261.63156	S	1074.46157	537.734
6	689.25420	345.13074	S-Phospho	987.42954	494.218
7	786.30696	393.65712	P	820.43118	410.719
8	843.32843	422.16785	G	723.37841	362.192
9	900.34989	450.67858	G	666.35695	333.682
10	999.41830	500.21279	V	609.33549	305.171
11	1162.48163	581.74445	Y	510.26707	255.637
12	1233.51875	617.26301	A	347.20374	174.105
13	1334.56642	667.78685	T	276.16663	138.586
14			R	175.11895	88.063

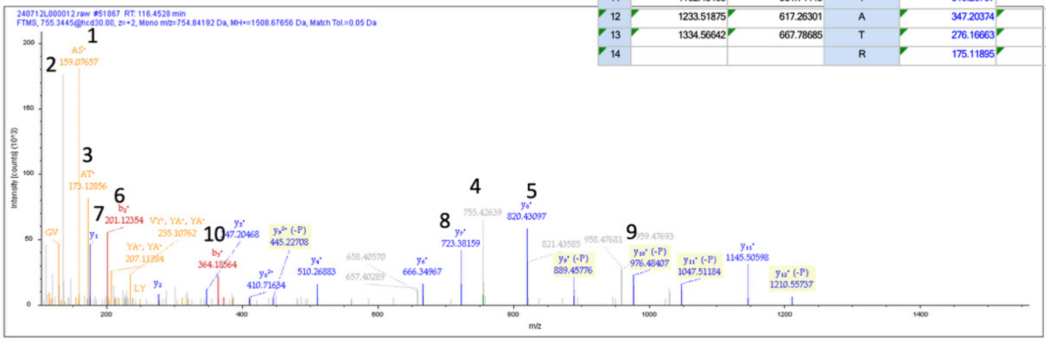

Supplementary Figure S4: Spectra of phospho-peptides having more than 1 peptide-spectral match and at least one unphosphorylated peptide mapping to the same protein. Numbers 1-10 on the spectra denote the top 10 most intense peaks on the spectra. Cross next to the protein name the peptides are derived from, indicates the spectra failed the quality control. Table shows in color which ions of the peptide sequence were annotated.

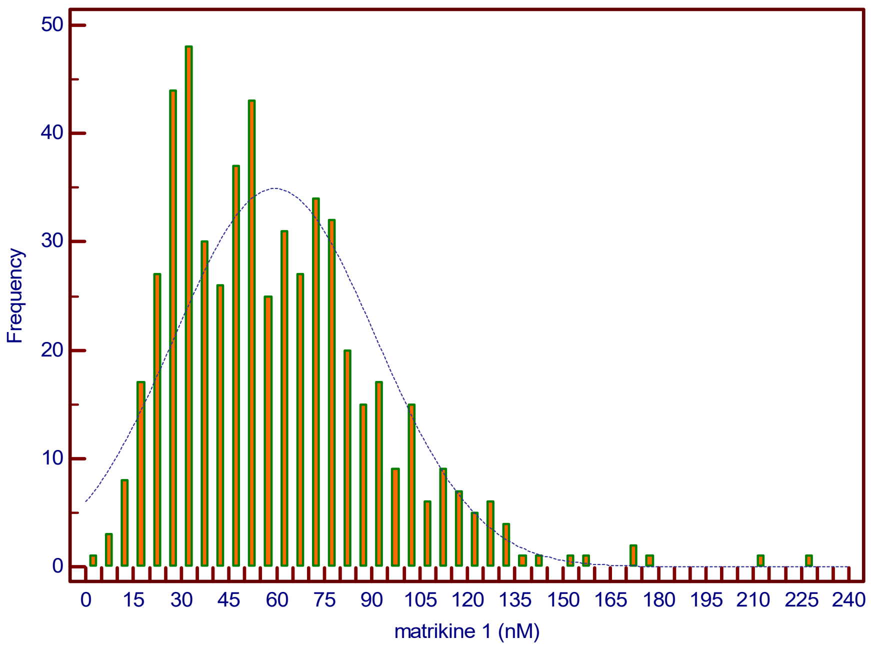

Supplementary Figure S5: Distribution of the concentration of peptide 1 in nM in 555 urine samples from individuals from the general population.
